# Quorum Quenching of *Nitrobacter winogradskyi* Suggests that Quorum Sensing Regulates Fluxes of Nitrogen Oxide(s) during Nitrification

**DOI:** 10.1128/mBio.01753-16

**Published:** 2016-10-25

**Authors:** Brett L. Mellbye, Andrew T. Giguere, Peter J. Bottomley, Luis A. Sayavedra-Soto

**Affiliations:** a Department of Botany and Plant Pathology, Oregon State University, Corvallis, Oregon, USA; b Department of Crop and Soil Science, Oregon State University, Corvallis, Oregon, USA; c Department of Microbiology, Oregon State University, Corvallis, Oregon, USA

## Abstract

Quorum sensing (QS) is a widespread process in bacteria used to coordinate gene expression with cell density, diffusion dynamics, and spatial distribution through the production of diffusible chemical signals. To date, most studies on QS have focused on model bacteria that are amenable to genetic manipulation and capable of high growth rates, but many environmentally important bacteria have been overlooked. For example, representatives of proteobacteria that participate in nitrification, the aerobic oxidation of ammonia to nitrate via nitrite, produce QS signals called acyl-homoserine lactones (AHLs). Nitrification emits nitrogen oxide gases (NO, NO_2_, and N_2_O), which are potentially hazardous compounds that contribute to global warming. Despite considerable interest in nitrification, the purpose of QS in the physiology/ecology of nitrifying bacteria is poorly understood. Through a quorum quenching approach, we investigated the role of QS in a well-studied AHL-producing nitrite oxidizer, *Nitrobacter winogradskyi*. We added a recombinant AiiA lactonase to *N. winogradskyi* cultures to degrade AHLs to prevent their accumulation and to induce a QS-negative phenotype and then used mRNA sequencing (mRNA-Seq) to identify putative QS-controlled genes. Our transcriptome analysis showed that expression of *nirK* and *nirK* cluster genes (*ncgABC*) increased up to 19.9-fold under QS-proficient conditions (minus active lactonase). These data led to us to query if QS influenced nitrogen oxide gas fluxes in *N. winogradskyi*. Production and consumption of NO_x_ increased and production of N_2_O decreased under QS-proficient conditions. Quorum quenching transcriptome approaches have broad potential to identify QS-controlled genes and phenotypes in organisms that are not genetically tractable.

## IMPORTANCE

Bacterial cell-cell signaling, or quorum sensing (QS), is a method of bacterial communication and gene regulation that is well studied in bacteria. However, little is known about the purpose of QS in many environmentally important bacteria. Here, we demonstrate quorum quenching coupled with mRNA-Seq to identify QS-controlled genes and phenotypes in *Nitrobacter winogradskyi*, a nitrite-oxidizing bacterium. Nitrite oxidizers play an important role in the nitrogen cycle though their participation in nitrification, the aerobic oxidation of ammonia to nitrate via nitrite. Our quorum quenching approach revealed that QS influences production and consumption of environmentally important nitrogen oxide gases (NO, NO_2_, and N_2_O) in *N. winogradskyi*. This study demonstrated a novel technique for studying QS in difficult-to-work-with microorganisms and showed that nitrite oxidizers might also contribute to nitrification-dependent production of nitrogen oxide gases that contribute to global warming.

## INTRODUCTION

Quorum sensing (QS) is a widespread process that bacteria use to coordinate gene expression with cell density, diffusion dynamics, and spatial distribution through the production of diffusible chemical signals (1, 2). Generally, as the density of bacterial cells increases, so does the concentration of QS signal, leading to coordinated expression of various genes in the entire bacterial population (1). The phenotypes associated with QS-controlled genes are often cooperative and stress-associated behaviors that benefit a population of bacteria, for example, biofilm formation, nutrient acquisition, luminescence, conjugation, and adaptation to stationary phase (2–4). There has been considerable interest in the study of bacterial QS, both in model systems for studying social evolution and to better understand important microbial behaviors, such as pathogenesis (1).

To date, most studies on QS have focused on model bacteria that are amenable to genetic manipulation (1). Many well-designed studies have introduced null mutations in the QS signal synthase and/or signal receptor-transcriptional regulator genes to determine what genes and phenotypes are controlled by QS (1). While this approach has significantly increased our understanding, many other QS-proficient organisms that are not easily genetically tractable have not been thoroughly studied. Through the use of bioassays, mass spectrometry, genome sequencing, and metagenomics, putative chemical signals and QS genes have been identified in representatives of proteobacteria that participate in the process of nitrification (5–10).

During nitrification, diverse genera of chemolithotrophic bacteria and/or archaea oxidize ammonia (NH_3_) to nitrite (NO_2_^−^) and then to nitrate (NO_3_^−^) (11–14). Generally, NH_3_ is oxidized to NO_2_^−^ by ammonia oxidizers, including bacteria (AOB) and archaea (AOA), while NO_2_^−^ is oxidized to NO_3_^−^ by nitrite-oxidizing bacteria (NOB). Recently, complete oxidation of NH_3_ to NO_3_^−^ (comammox) was identified in representatives of the genus *Nitrospira*, a group previously characterized as NOB (15, 16). Nitrification is a key part of the nitrogen cycle in natural, agricultural, and industrial systems and is a contributor to gas emissions of nitric oxide (NO), nitrogen oxides (NO_x_), and nitrous oxide (N_2_O), which are hazardous gases that contribute to global warming (11, 17).

In many proteobacteria, QS is accomplished through the production of acyl-homoserine lactone (AHL) signaling compounds or autoinducers (1). AHLs represent the best-studied class of autoinducer, and they are generally produced by a LuxI homolog autoinducer synthase and detected by a LuxR homolog signal receptor-transcriptional regulator (1). The *luxI* and *luxR* genes are commonly located adjacent to each other in the genome and are generally positively autoregulated (1).

One method to study AHL QS is by specifically inactivating all AHL autoinducers through the use of recombinant lactonase to promote a QS-deficient phenotype (18, 19). AiiA, an AHL lactonase identified from *Bacillus* spp., is a well-characterized enzyme that specifically hydrolyzes the homoserine lactone (HSL) ring of AHLs, regardless of the chain length of the acyl group or other moiety (20, 21). So-called “quorum quenching” approaches have been implemented through both heterologous expression in a host of interest and addition of purified AHL lactonase (18, 20, 22–24). In this study, we used purified AiiA lactonase to identify QS-controlled gene expression and phenotypes in *Nitrobacter winogradskyi*, a well-characterized NOB that is currently not genetically tractable.

The genus *Nitrobacter* consists of a ubiquitous group of NOB in the family *Bradyrhizobiaceae* isolated from soil, water, and wastewater treatment systems (13, 14). *N. winogradskyi* is a well-studied example of NOB due to its superior growth rate and growth yield compared to other NOB, and it was the first NOB shown to produce AHLs (10, 13, 14). In addition, the genome of *N. winogradskyi* has been sequenced and this bacterium has been the subject of recent global transcriptome studies (6, 25, 26). Expression of *N. winogradskyi* genes *nwiI*, encoding an autoinducer synthase, and *nwiR*, encoding a receptor-transcriptional regulator, was shown to be cell density dependent and to correlate with the AHL concentration in culture (10). The structure of the predominant AHL was identified as that of an unsaturated AHL with a 10 carbon acyl chain, C_10:1_-HSL (10). Nuclear magnetic resonance (NMR) spectroscopy analysis of AHL extracts produced via heterologous expression in *Escherichia coli* identified the isomeric form of C_10:1_-HSL (27) but suggested a location for the double bond that is different from that previously described (10). However, heterologous expression of autoinducer synthases in *E. coli* often produces AHLs that are different from those in the native strain (27, 28).

Previous attempts have been made to identify QS-controlled phenotypes in *N. winogradskyi* (10, 27). Mellbye et al. showed that the growth rate decreased as transcription of *nwiI* and *nwiR* increased and AHLs began to accumulate (10). Shen et al. observed up to a 2-fold increase or 5-fold decrease in the expression of select genes of the nitrite oxidoreductase (NXR) gene cluster after the addition of purified C_10:1_-HSL to cultures at saturating concentrations but did not observe any statistically significant phenotypic changes (27).

Here, we utilized a quorum quenching approach to identify both primary and secondary regulatory effects of AHL QS in *N. winogradskyi*. Using purified AiiA lactonase, AHLs were depleted from *N. winogradskyi* cultures and QS-controlled genes were identified through comprehensive mRNA sequencing (mRNA-Seq) analysis. Our transcriptome analysis showed that depletion of AHLs affected the expression of a significant percentage (52%) of the genetic inventory in *N. winogradskyi* and also suggested a link between QS and nitrogen oxide fluxes in this bacterium. Our experiments confirm a previous report that *N. winogradskyi* can produce N_2_O (29) and present new evidence that QS affects NO_x_ fluxes. Our work demonstrates that AiiA-mediated quorum quenching coupled with mRNA-Seq is a useful technique to identify QS-controlled genes and phenotypes in difficult-to-study organisms.

## RESULTS

### AiiA lactonase treatment of *N. winogradskyi* cultures depletes AHLs.

**FIG 1:**
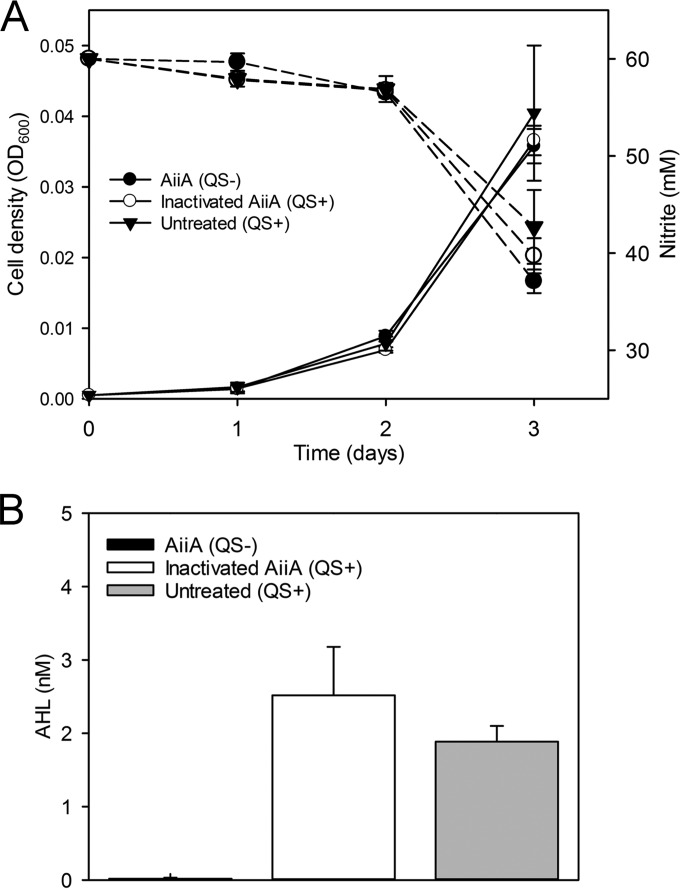
Batch culturing comparison of *N. winogradskyi* results determined under QS-proficient and -deficient conditions. (A) Closed circles represent AiiA lactonase-treated (QS-deficient) cultures, open circles represent heat-inactivated AiiA lactonase (QS-proficient) cultures, and triangles represent untreated (QS-proficient) cultures. Solid lines correspond to cell density measured as the optical density at 600 nm (OD_600_; left *y* axis), and dashed lines correspond to the NO_2_^−^ concentration in millimolar (right *y* axis) measured over time (days; *x* axis). (B) Bars indicate AHL concentrations in nanomolar (*y* axis) of AiiA lactonase-treated (QS-deficient) cultures, heat-inactivated AiiA lactonase-treated (QS-proficient) cultures, and untreated cultures (QS-proficient) when cultures were harvested on day 3. Depending on cell density, approximately 0.28 or 0.71 µg protein ml^−1^ was added to both lactonase and heat-denatured lactonase treatments daily (see Text S1 in the supplemental material). Values are the means of the results of four independent biological replicates. Error bars indicate the standard deviations of the means.

To determine the effect of QS inhibition in *N. winogradskyi*, we initiated and monitored three batch culture treatments: (i) AiiA lactonase treatment (QS-deficient), (ii) heat-denatured AiiA lactonase treatment (QS-proficient) (to determine if protein addition had an effect), and (iii) no-added-lactonase treatment (QS-proficient). Depending on cell density, approximately 0.28 or 0.71 µg protein ml^−1^ was added to both lactonase and heat-denatured lactonase treatments daily (see Text S1 in the supplemental material). Although the treatments showed no significant differences in nitrite oxidation rate, growth rate, or growth yield (Fig. 1A), the addition of AiiA lactonase prevented the accumulation of bioassay-detectable AHL (Fig. 1B). Lactonase-treated and heat-denatured lactonase-treated cultures were harvested on day 3 during peak signal production as observed in our previous work (10) to collect RNA for mRNA-Seq (Fig. 1).

### Transcriptome responses to QS inhibition.

**FIG 2:**
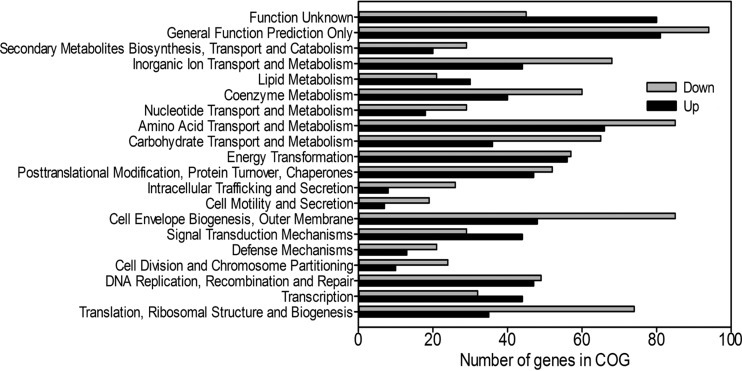
Clusters of orthologous group (COG) assignments of gene expression under QS-proficient conditions. Bars indicate the number of genes with increased expression (black) or the number of genes with decreased expression (gray) under QS-proficient conditions for each functional group. A quantity of 100 genes corresponds to 3.2% of COG assignments in the genome. In total, 56.3% of the COG assignments changed in expression level. Expression changes correspond to the difference in mRNA transcript levels between AiiA-treated QS-deficient cells and QS-proficient cells (*P* ≤ 0.05).

The transcriptome of *N. winogradskyi* under QS-deficient (AiiA-treated) conditions was compared to that present under QS-proficient (heat-denatured AiiA-treated) conditions. All changes in gene expression are expressed as the ratio of the number of transcripts seen under the QS-proficient treatment conditions to the number seen under QS-deficient treatment conditions. First, we validated our quorum quenching approach by noting an increase in the transcript abundance of the signal synthase *nwiI* gene and the signal receptor *nwiR* gene under QS-proficient conditions (Table 1). As previously noted, many bacterial QS genes, particularly the signal synthase gene, are autoregulated, creating a positive-feedback loop (1, 10). In addition, levels of methionine biosynthesis transcripts increased up to 7.7-fold, possibly due to increased use of *S*-adenosyl methionine for AHL biosynthesis (Table 1). The transcriptome analysis revealed 1,631 genes showing statistically significant changes in expression in QS-proficient cells, but many changes were <3-fold (see Table S1 and Dataset S1 in the supplemental material). In total, expression of 1,346 genes changed marginally and expression of 237 genes changed >3-fold between QS-deficient and QS-proficient conditions (see Dataset S1). Grouping the expression changes into clusters of orthologous groups (COG) functional categories, COGs associated with the process of translation as well as nucleotide, carbohydrate, and amino acid metabolism and transport substantially (>60% of category COGs) changed in expression (Fig. 2). We observed a similar trend whether an expression cutoff (e.g., 2-fold cutoff) was applied or not; thus, all of the data were included in the COG analysis.

**TABLE 1:** QS-dependent changes in gene expression in *N. winogradskyi*

Gene category or number(s)	Gene name(s)	Description or role	Fold change^a^
Quorum sensing			
Nwi0283, Nwi0284, Nwi0403, Nwi0586, Nwi2890	*metH*, *metW*	Methionine biosynthesis	1.5 to 7.7
Nwi0626	*nwiI*	Autoinducer synthase	2.5
Nwi0627	*nwiR*	AHL-binding LuxR	1.3
Biosynthetic metabolism			
Nwi0719, Nwi0720	*nirBD*	Assimilatory nitrite reductase	−2.5 to −9.3
Nitrogen metabolism			
Nwi2648	*nirK*	Putative nitrite reductase, NO production/consumption	2.2
Nwi2653–Nwi2649	*ncgABC*	*nirK* cluster genes, NO production/consumption	2.7 to 19.9
NO and/or guanine nucleotide signaling			
Nwi0500		Diguanylate cyclase/phosphodiesterase	3.7
Nwi0529, Nwi0597–Nwi0599, Nwi1111, Nwi1121–Nwi1124, Nwi1130, Nwi1132–Nwi1134	*flhA*, *fliH*, *fliG fliF*, *flgI*, *flgG*, *flgF*, *fliL*, *fliM*, *fliP*, *flgB*, *flgC*, *fliE*	Flagellum biosynthesis/assembly	−1.3 to −2.6
Nwi0557	*nnrS*	NO-related gene product	8.1
Nwi1922		RelA/SpoT homolog	2.3
Nwi2061		Crp domain regulator	8.9
Nwi2151		Ppx/GppA phosphatase	3.2

a Fold change data correspond to the difference in mRNA transcript levels between AiiA-treated QS-deficient cells and QS-proficient cells (*P* ≤ 0.05, *n* = 4).

Based on previous work that suggested that QS is autoregulated in *N. winogradskyi* (10), we used SCOPE to search for *lux*-box-like inverted repeat motifs upstream of *nwiI* and *nwiR* (see Text S1 and Fig. S2 in the supplemental material) (30). Two different motifs (motif A and motif B) were identified that suggested that QS directly activates *nirK* cluster genes, as well as the stringent response secondary messenger system mediated through GppA phosphatase, and expression of several other genes (see Fig. S1 and Table S2).

### Nitrite metabolism and signal transduction genes are induced under QS-proficient conditions.

An in-depth scan of the QS transcriptome of *N. winogradskyi* showed that the largest changes in expression involved genes encoding proteins associated with biosynthetic metabolism, nitrogen metabolism, and signal transduction, particularly those associated with nitrite metabolism (Table 1). Under QS-proficient conditions, assimilatory nitrite reductase gene *nirBD* decreased in expression up to 9.3-fold, suggesting imminent growth arrest and induction of the stringent response, as nitrite was the sole nitrogen source in the medium (Table 1). In addition, expression of nitrite reductase gene *nirK* increased 2.2-fold whereas expression of *nirK* cluster genes, including *ncgABC*, increased up to 19.9-fold under QS-proficient conditions (Table 1). Furthermore, expression of Nwi0557, a homolog of *nnrS*, a putative NO-responsive membrane protein gene, increased 8.1-fold (Table 1). These changes suggest a possible link between QS effects on N biosynthesis metabolism and NO_x_ metabolism.

QS-proficient conditions also changed the expression levels of several genes involved in signal transduction and flagellum biosynthesis. Guanine nucleotide secondary messenger (e.g., ci-di-GMP, ppGpp) biosynthesis and response genes, including genes encoding a diguanylate cyclase/phosphodiesterase homolog (Nwi0500), a Crp domain regulator (Nwi2061), a RelA/SpoT homolog (Nwi1922), and Ppx/GppA phosphatase (Nwi2151), increased in expression by 2.3- to 8.9-fold (Table 1). Fourteen genes associated with flagellum biosynthesis and assembly decreased in expression up to 2.6-fold (Table 1). That said, no obvious phenotype differences, such as changes in motility, biofilm, or aggregate formation, were observed under QS-proficient or -deficient conditions.

### Quorum sensing in *N. winogradskyi* influences NO_x_ fluxes.

Following the transcriptome analysis prediction that QS affects nitrite metabolism through expression of *nirK* cluster genes, production and consumption of NO_x_ gases by *N. winogradskyi* were measured. In order to observe the biggest difference in gas fluxes, cells were incubated at high cell density in sealed vials conducive to AHL accumulation. Preliminary tests detected abiotic NO_x_ accumulation in the headspace above sterile nitrite-containing growth medium, most likely due to the aqueous chemical decomposition of protonated NO_2_^−^ (nitrous acid [HNO_2_; also known as HONO]) to NO and NO_2_, collectively referred to as NO_x_ gases (31). Therefore, we included both heat-killed cells and sterile medium controls along with our QS-proficient and -deficient treatments in the experiments. NO_x_ gas measurements were made from such suspensions of concentrated QS-proficient or -deficient *N. winogradskyi* cells during 24 h of NO_2_^−^ oxidation. We predicted that an increase in expression of the *nirK* cluster genes under QS-proficient conditions would either increase NO_x_ production as earlier studies have suggested (32–34) or decrease NO_x_ production by consuming NO as previously reported (34–36).

**FIG 3:**
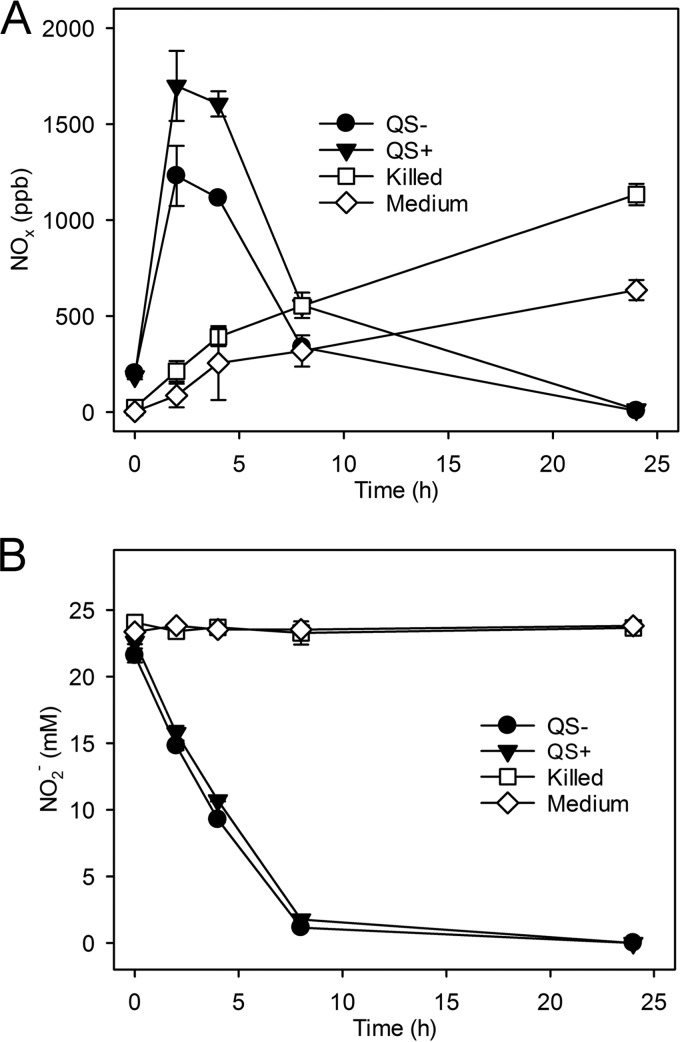
QS-dependent production and consumption of NO_x_ gases in *N. winogradskyi*. Closed circles (QS−) indicate AiiA lactonase-treated (QS-deficient) cells, closed triangles (QS+) indicate untreated cells (QS-proficient), open squares (killed) indicate heat-inactivated cell controls, and open diamonds (medium) indicate sterile medium controls. All measurements were made over 24 h (*x* axis). (A) Values correspond to NO_x_ gases accumulated in the headspace measured as parts per billion (ppb; *y* axis). (B) Values show NO_2_^−^ concentrations (millimolar; *y* axis) in solution. Values are the means of the results of four independent biological replicates. Error bars indicate the standard deviations of the means.

A statistically significant (*P* < 2 × 10^−6^) accumulation of NO_x_ (measured as parts per billion [ppb] by volume) was registered for both QS-proficient and -deficient treatments (approximately 1,699 and 1,240 ppb, respectively) after 2 h of incubation, compared to the accumulations seen with medium alone and with heat-killed cell controls (approximately 87 and 213 ppb, respectively) (Fig. 3A). The peak NO_x_ accumulation in both the QS-proficient and -deficient treatments was transient and was followed by the disappearance of NO_x_ as NO_2_^−^ was consumed by *N. winogradskyi* (Fig. 3). In contrast, heat-killed cells and medium-alone controls slowly accumulated NO_x_ in the headspace over time and NO_2_^−^ concentrations did not change significantly (Fig. 3). The pH of *N. winogradskyi* cultures and controls did not change significantly during the experiments (data not shown). Although these data suggest that some abiotic NO_x_ accumulation occurred in the controls without *N. winogradskyi* cells, transient NO_x_ production occurring during active NO_2_^−^ oxidation by *N. winogradskyi* was significantly greater than that seen with the controls. QS-proficient cells both produced and consumed NO_x_ at significantly greater rates (*P* < 0.003) than AiiA-treated, QS-deficient cells (Fig. 3A). Addition of heat-denatured AiiA to either growth medium or heat-killed cell controls did not affect the rate of NO_x_ accumulation or consumption (data not shown). QS-proficient cells produced approximately 756 ppb NO_x_ h^−1^ during the initial 2 h of the experiment, while QS-deficient cells produced 514 ppb NO_x_ h^−1^ (Fig. 3A). Between h 4 and h 8, the net levels of consumption of NO_x_ by QS-proficient and QS-deficient cells were 262 ppb h^−1^ and 191 ppb h^−1^, respectively (Fig. 3A).

### QS inhibition increases N_2_O production by *N. winogradskyi*.

**FIG 4:**
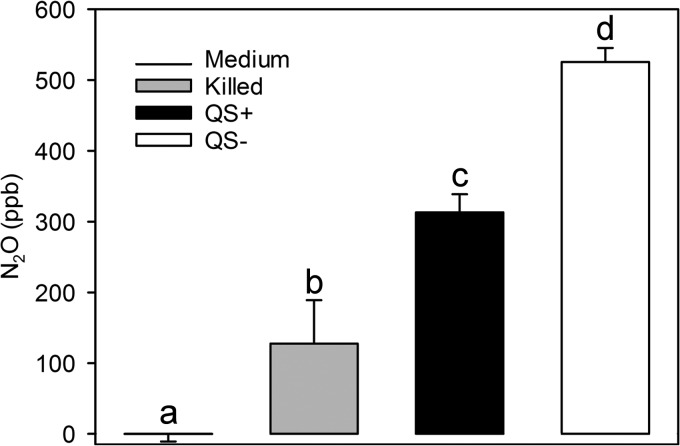
QS-dependent production of N_2_O by *N. winogradskyi*. N_2_O accumulation in headspace is shown as parts per billion above atmospheric N_2_O (ppb; *y* axis). The dark line (medium) indicates medium controls, the light gray bar (killed) indicates heat-inactivated cell controls, the black bar (QS+) indicates untreated QS-proficient cells, and the white bar (QS−) indicates AiiA lactonase-treated QS-deficient cells. Values are the means of the results of four independent biological replicates. Error bars indicate the standard deviations of the means. Different letters represent significant differences between treatments determined by a one-way analysis of variance (*P* < 0.0001, *n* = 4).

Following a previous unsubstantiated report of N_2_O production by *Nitrobacter* species (29), we measured N_2_O accumulation by both QS-proficient and -deficient concentrated *N. winogradskyi* cells after 24 h of NO_2_^−^ oxidation. Despite the absence of a known nitric oxide reductase gene (*nor*) in the *N. winogradskyi* genome, *N. winogradskyi* cells accumulated significantly (*P* < 0.002) more N_2_O than either growth medium alone or heat-killed cell controls (Fig. 4). AiiA-treated, QS-deficient cells accumulated approximately 525 ppb N_2_O, 1.68-fold more N_2_O than QS-proficient cells (313 ppb N_2_O) (*P* < 0.0001) (Fig. 4). In addition, the molar ratio of NO_x_-N at 2 h to normalized N_2_O-N accumulated at 24 h (after all NO_x_ was consumed) was considerably higher (6.90 NO-N/N_2_O-N ratio) than that seen under QS-deficient conditions (2.78 NO-N/N_2_O-N ratio). These data suggest that QS also affects N_2_O production by *N. winogradskyi*, likely through changes in NO_x_ fluxes.

## DISCUSSION

### Quorum quenching transcriptomics is a novel technique to identify QS-controlled genes and phenotypes.

We used a quorum quenching transcriptomic technique to investigate the role of QS in *N. winogradskyi*, a nitrite-oxidizing bacterium that participates in the nitrogen cycle. Our experiments identified a QS-controlled phenotype and revealed a link between QS and NO_x_ metabolism in a nitrifying microorganism. While mutational analysis is the preferred method to study QS, there are many bacteria, including those of environmental importance, with no established genetic system and new techniques are needed to determine the purpose of QS in these organisms. Quorum quenching also represents a different methodology to study animal pathogens and social evolution (20, 22–24). Mutant construction in most bacteria takes several generations of selection that may introduce unintended changes, particularly during social-evolution experiments. As previously suggested, quorum quenching through addition of a lactonase or chemical inhibitor is a quicker method to induce a QS-deficient phenotype without the need for several generations of selection and the possibility of pleiotropic effects (23, 24, 37).

### NO_x_ metabolism in *N. winogradskyi*.

NO production and consumption in *N. winogradskyi* have generated considerable interest and confusion for almost 30 years. Earlier work by Freitag et al. and Freitag and Bock suggested that *Nitrobacter* strains consume NO to generate NADH and produce N_2_O under various conditions (29, 35). Starkenburg et al. published the sequenced genome of *N. winogradskyi* and noted that it lacked the gene(s) known to produce N_2_O but possessed a putative NO-producing gene, *nirK*, and *ncgABC*, closely related to homologs in *N. europaea* (6). However, later work by Starkenburg et al. could not confirm production of NO by *N. winogradskyi* when *nirK* was expressed but did show that *N. winogradskyi* was able to consume NO (36). Coculture studies of the AOB *Nitrosomonas europaea* and *N. winogradskyi* suggest that *N. winogradskyi* may consume NO, as less NO accumulated during coculture but expression of *nirK* in *N. winogradskyi* increased (25, 38). Finally, the studies described above and work on other NOB and AOB were incorporated into a multispecies metabolic model to assess sources and sinks of NO in relation to N_2_O production during wastewater treatment (34). The model predicts that NOB likely oxidize NO to NO_2_^−^ but do not substantially contribute to N_2_O production (34).

Our quorum quenching transcriptomics approach led us to investigate production and consumption of NO_x_ gases in *N. winogradskyi* and showed that *N. winogradskyi* may function as a source and/or a sink of NO_x_ and N_2_O. We hypothesized that if increased expression of *nirK* and associated genes under QS-proficient conditions increased the concentration of NO_x_ gases, then more NO would be available for abiological or nonspecific mechanism-based reduction to N_2_O. Contrary to our prediction, QS-deficient cells produced significantly more N_2_O than QS-proficient cells. A closer inspection of the molar ratio of peak NO_x_-N and N_2_O-N produced shows that QS-proficient cells not only produced more NO_x_ than QS-deficient cells but also directed considerably less of the NO_x_ to the N_2_O pool than QS-deficient cells. These data possibly suggest consumption of NO_x_ via oxidation back to nitrite and, subsequently, nitrate, as previously suggested (36), or that there are alternate fates for NO such as unspecified signaling roles.

Note that *N. winogradskyi* produced considerably less N_2_O than NO, as previously predicted (34). *N. winogradskyi* does not contain a known nitric oxide reductase (nor), and, while our data show that some of the N_2_O formation is dependent on live cells, there may be abiotic reactions of NO with cellular components as has been recently suggested during ammonia oxidation by *Thaumarchaeota* (39). This hypothesis may partially explain why QS-deficient cells produce more N_2_O, since their consumption of NO_x_ is slower than that by QS-proficient cells.

Considering the close homologies between *nirK* and *ncgABC* of *N. winogradskyi* and *N. europaea*, the two gene clusters may serve similar purposes. In *N. europaea*, both *nirK* and *ncgABC* were shown to confer tolerance to NO_2_^−^, but *nirK* had a negative fitness effect in *ncgABC* mutants (40, 41). Our observation of an up to 19.9-fold increase in *ncgABC* transcripts (Table 1; see also Table S1 and Dataset S1 in the supplemental material) under QS-proficient conditions suggests that these genes may play a role in NO_x_ consumption or detoxification in *N. winogradskyi*, but future work is needed to investigate this prediction.

One interesting observation arising from our work on NO_x_ flux is the abiotic generation of NO_x_ from NO_2_^−^, likely via chemical decomposition of aqueous HNO_2_ or of its gaseous analog, HONO. Although this phenomenon was studied extensively in the past (31), reviewed in the nitrification and engineering fields (33), and recently appreciated in soils (42, 43), most studies in the nitrification and environmental engineering fields have still largely ignored it. Many studies on nitrifying microorganisms, particularly NH_3_ oxidizers, routinely measure NO but do not account for NO_x_ generated abiotically from the NO_2_^−^ end product of NH_3_ oxidation. In addition, many metabolic modeling studies, including an important study cited here modeling NO and N_2_O turnover (34), have not included abiotic formation of NO as a significant source. Clearly, future models and studies need to consider the contribution of abiotic reactions to NO_x_ production.

### Why does QS regulate NO_x_ metabolism?

The immediate rapid generation of NO by concentrated *N. winogradskyi* cells is an initially puzzling response for a NO_2_^−^ oxidizer, since reductant would be required to reduce NO_2_^−^ to NO. However, metabolic modeling of electron flow in *Nitrobacter* has suggested that generation and consumption of NO would help explain previous experimental data (32). Some support for QS regulation of this metabolic response recently emerged, as *Nitrobacter* accumulated fewer AHLs under mixotrophic than under autotrophic growth conditions, perhaps also suggesting less generation of NO when organic carbon is available (27). Another possible explanation is the model proposed by Starkenburg et al. that suggests that NO production is a strategy to reversibly block the terminal cytochrome oxidase and redirect electrons toward generation of reductant (36). This hypothesis would make sense if one role for QS is to promote redirection of electrons away from reductive cellular biosynthesis and toward generation of electron-rich storage compounds such as poly-β-hydroxybutyrate as previously reported (6).

In other bacteria, QS generally controls production of a public good that can be used by the entire population (1). According to previous reports that *Nitrobacter* generates NADH from NO oxidation (35), an increase in NO generation at higher cell densities (QS-proficient conditions) may function as a public good for energy generation. NO generated in a large population of *Nitrobacter* is more likely to be utilized by nearby *Nitrobacter* cells and may benefit the population.

Another possible function for QS regulation of the *nirK* cluster and other genes could be preparation for stationary phase, partially through NO signaling. Signal integration of QS and stress responses has been previously demonstrated in *Pseudomonas aeruginosa* and other bacterial species (3). Transcriptome data suggest that QS-proficient conditions both prepare for growth arrest via repression of assimilatory nitrite reductase *nirBD* and induction of the stringent response and promote transition from a motile to sessile state through inhibition of flagellar expression and possible guanine secondary messenger signaling (Table 1). Many of these and other changes in transcription could be indirect effects of NO signaling. According to transcriptome data, QS activation triggers transcription of a putative *nnrS* homolog (Table 1). While the role of *nnrS* in *N. winogradskyi* is unknown, this gene was previously shown to be transcribed during exposure to NO and to regulate chemotaxis in *Rhodobacter sphaeroides* (44, 45). NnrS has recently been proposed to function as an NO sensor (46), and we speculate that it may serve a similar role in *N. winogradskyi*. NO signaling would also be a convenient way to detect nearby AOB, since some NO is produced via NH_3_ oxidation.

The identification of QS regulation of NO_x_ metabolism in *N. winogradskyi* raises questions about NO_x_ metabolism in other NOB. A cursory search of genomic databases shows that all NOB and comammox bacteria contain *nirK* homologs and that all except *Nitrococcus* and *Nitrolancea* contain *nnrS* homologs but that only *Nitrobacter* species contain both *ncgABC* and clearly annotated autoinducer synthase and receptor genes associated with QS. Since *Nitrobacter* species are *r*-strategists with the ability to exploit higher substrate concentrations and sporadically grow to higher densities, they may make better use of cell-density-dependent QS genetic regulation (10, 47). In addition, as *r*-strategists, *Nitrobacter* species might use QS-controlled preparation for starvation as an important strategy to recognize transitions to an unfavorable energy-limited situation (48). Future research into *nirK* function in NOB is needed to confirm the role of *nirK* and *nnrS* in NO_x_ fluxes and to determine if NO signaling occurs in these microorganisms.

## MATERIALS AND METHODS

### Chemicals.

*N*-Decanoyl-dl-homoserine lactone (C_10_-HSL) was purchased from Sigma-Aldrich (St. Louis, MO). Acetic acid and high-performance-liquid-chromatography (HPLC)-grade ethyl acetate were purchased from VWR International (Radnor, PA) and EMD Chemicals (Darmstadt, Germany), respectively.

### Bacterial strains, plasmids, and growth medium.

Bacterial strains and plasmids used in this study are outlined in Table S3 in the supplemental material. *N. winogradskyi* was routinely cultivated in 60 mM NaNO_2_-supplemented mineral salts medium as described previously (26), with minor modifications for NO_x_ and N_2_O measurements (see Text S1 in the supplemental material and the descriptions of AiiA QS inhibition and culturing experiments below). *N. winogradskyi* cultures were routinely screened for heterotrophic contamination by plating 200-µl aliquots of culture on Luria-Bertani (LB) agar plates. *Agrobacterium tumefaciens* was prepared and cultivated as described elsewhere (49, 50). *Escherichia coli* strains were grown in LB medium on a rotatory shaker at 200 rpm and 37°C.

### AiiA lactonase production and activity measurement.

Plasmid pDSK519 carrying *aiiA* was kindly provided by Max Teplitski and Mengsheng Gao of the University of Florida. The *aiiA* gene was cloned, and AiiA was expressed and purified as outlined in Text S1 in the supplemental material. AiiA-specific activity units were determined by measuring reductions of AHL concentrations after 4 hours as outlined in Text S1 in the supplemental material.

### AHL bioassay.

AHLs were extracted from *N. winogradskyi* cultures and quantified in Miller units by broad-range *Agrobacterium tumefaciens* bioassay as described previously (10, 49, 50). AHL concentrations (nanomolar) were estimated using standard concentrations of C_10_-HSL (see Fig. S2 in the supplemental material). For determining AiiA activity, 200 µl of the assay solution was directly added to *A. tumefaciens* culture as described previously (49, 50).

### AiiA QS inhibition and culturing experiments.

Batch cultures of *N. winogradskyi* were prepared in 100 ml 60 mM NO_2_^−^-supplemented medium at pH 7.5 as outlined above, inoculated to an optical density at 600 nm (OD_600_) of 0.001 from mid-exponential-phase cultures, and grown in Erlenmeyer flasks on a rotatory shaker at 100 rpm and 30°C. All experiments, including mRNA-Seq experiments, included 4 biological replicates. For NO_x_ and N_2_O measurements, batch cultures were either treated with AiiA lactonase as outlined in Text S1 in the supplemental material or left untreated and were harvested by centrifugation. Harvested cells were suspended to an OD_600_ of 0.2 in 25 mM (NO_x_ measurement) or 60 mM (N_2_O measurement) NO_2_^−^-supplemented medium and treated with AiiA lactonase or left untreated or subjected to heat killing by incubation at 110°C for 20 min. Cultures were placed in 41-ml serum vials. The serum vial cultures were then capped with gray-butyl stoppers, crimp sealed, and incubated for 24 h as outlined. NO_x_ and N_2_O levels were routinely measured in the headspace as outlined below. Experimental cultures were routinely monitored every 24 h to check cell density (OD_600_), NO_2_^−^ concentration by the Griess assay (51), and AHL concentration as described above.

For QS inhibition transcriptome experiments and other experiments, a specific number of activity units of AiiA lactonase (filtered using a 0.2-µm-pore-size filter) was added into *N. winogradskyi* batch cultures every 24 h (QS-deficient conditions) as outlined in Text S1 in the supplemental material.

### RNA preparation and sequencing.

RNA was extracted using an RNeasy minikit (Qiagen, Germantown, MD) (10), and mRNA was enriched and prepared for Illumina mRNA-Seq as described previously (52). The libraries were sequenced (150mer paired-end sequencing) on a HiSeq 3000 Sequencer (Illumina, San Diego, CA) at the Center for Genome Research and Biocomputing Core Laboratories at Oregon State University.

### Transcriptome data analysis.

The mRNA-Seq data were analyzed using the CLC Genomics Workbench (CLC bio, Prismet, Denmark) as previously described (52). Briefly, mRNA-Seq reads were normalized to reads per kilobase of transcript per million mapped reads (RPKM) and the module for empirical analysis of differential gene expression (DGE) was used as described previously (53, 54). Quantitative reverse transcription-PCR (qRT-PCR) was used to corroborate gene expression of selected genes with total RNA from biological replicates and primers as described in Text S1 in the supplemental material and outlined in Table S4 and Table S5.

### Analytical methods.

NO_2_^−^ levels were determined by chemical assay as described previously (51). NO_x_ levels were measured using a portable NO_x_ detector (LMA-3D and LNC-3D; Unisearch Associates Ltd., Concord, Ontario, Canada) that passes air through a CrO_3_ filter to convert NO to NO_2_ and then measures parts per billion by volume of NO_2_ by chemiluminescence as described previously (55), with modifications. Briefly, 1 ml or 5 ml of samples was injected into the intake line of the instrument and the peak NO_x_ level recorded. NO_x_ peaks were quantified by comparison to both NO and acidified NO_2_^−^ standards. N_2_O was measured by gas chromatography as described previously (52). Cell density was measured as OD_600_, and the protein concentration was measured with a Pierce bicinchoninic acid (BCA) protein assay kit (Thermo Scientific, Rockford, IL).

### Accession number(s).

Raw datasets and processed datasets are available at Gene Expression Omnibus (GEO) at the National Center for Biotechnology Information (NCBI) under accession no. GSE84969.

## SUPPLEMENTAL MATERIAL

Text S1  Supplemental Materials and Methods: detailed methods, including medium formulation, promoter element prediction, corroboration of mRNA-Seq results by qRT-PCR, AiiA purification, activity measurement, and QS inhibition. Download Text S1, PDF file, 0.1 MB

Dataset S1  mRNA-Seq expression data generated by Illumina HiSeq 3000 Sequencer (Illumina, San Diego, CA) and analyzed using the CLC Main Workbench (CLC bio, Prismet, Denmark). Fold change data indicate changes in expression between AiiA-treated, QS-deficient cells and QS-proficient cells treated with heat-inactivated AiiA. Download Dataset S1, XLSX file, 1 MB

Figure S1  Putative *lux*-box-like promoter elements in *N. winogradskyi*. Sequence logos shown are graphic representations of aligned sets of putative promoter DNA sequences displaying frequencies of bases at each position as the relative heights of letters. The sequence logo also shows the degree of sequence conservation as the total height of a stack of letters, measured in bits. (a) Promoter motif A found upstream of *nwiRI*, the *nirK* cluster, and other genes. (b) Promoter motif B found upstream of *nwiRI*, the GppA phosphatase gene, and other genes. Table S2 shows all genes with these promoter motifs. Download Figure S1, TIF file, 0.2 MB

Figure S2  *N*-Decanoyl-l-homoserine lactone (C_10_-HSL) acyl-homoserine lactone (AHL) bioassay standard curve. Known concentrations of C_10_-HSL were added to bioassay cultures (see Materials and Methods in the main text) for estimation of the concentration of AHLs in *N. winogradskyi* cultures. The black line corresponds to the nonlinear regression of C_10_-HSL concentration (nanomolar, *y* axis) compared to AHL bioassay Miller units (*x* axis), and dotted lines indicate the 95% confidence band. The regression was calculated as follows: *y* = 0.0047*x* + 5 × 10^− 5^*x*^2^, *R*^2^ = 0.99. Download Figure S2, TIF file, 0.2 MB

Table S1  Statistically significant changes in gene expression under QS-proficient conditions in *N. winogradskyi* Table S1, PDF file, 0.2 MB

Table S2  Putative QS-controlled genes with upstream *lux*-box-like promoter elements. Table S2, PDF file, 0.1 MB

Table S3  Bacterial strains and plasmids. Table S3, PDF file, 0.1 MB

Table S4  Comparison of fold changes in expression between QS-deficient and proficient treatments analyzed by quantitative PCR (qPCR) and mRNA-Seq. Table S4, PDF file, 0.1 MB

Table S5  Genes and primers used to corroborate gene expression. Table S5, PDF file, 0.1 MB
