# Supplementary material for: Quorum Quenching of Nitrobacter winogradskyi Suggests that Quorum Sensing Regulates Fluxes of Nitrogen Oxide(s) during Nitrification
Source: mBio. 2016 Oct 25;7(5):e01753-16. doi: 10.1128/mBio.01753-16 (PMC5080386; doi:10.1128/mBio.01753-16)
Supplement: Text S1 — Supplemental Materials and Methods: detailed methods, including medium formulation, promoter element prediction, corroboration of mRNA-Seq results by qRT-PCR, AiiA purification, activity measurement, and QS inhibition. Download [file mbo005163044s1.pdf]

Supplementary Materials and Methods.

Medium formulation.

Nitrobacter winogradskyi was routinely cultivated in mineral salts medium consisting of either 60 mM $NaNO_2$ or 25 mM $NaNO_2$, 0.75 mM $MgSO_4$, 0.2 mM $CaCl_2$, 4.1 mM K_2HPO_4, 0.28 mM Na_2CO_3, and trace minerals: 9.9 μM $FeCl_3$, 10.0 μM $CuSO_4$, 0.6 μM $Na_2MoO_4 \cdot H_2O$, 1.59 μM $MnCl_2$, 0.6 μM $CoCl_2$, and 0.096 μM $ZnCl_2$ as previously described (1). The pH of the medium was adjusted to 7.5 with HCl.

Promoter element prediction.

Putative promoter elements under direct QS control were identified by the Suite for Computational identification Of Promoter Elements (SCOPE) (2). Briefly, upstream intergenic regions of *nwiI*, *nwiR*, and the most highly expressed QS-controlled gene cluster (Nwi2653-2648) were searched for inverted repeat motifs. Next, the entire transcriptome was searched for identified promoter motifs greater than or equal to 8 bases.

Corroboration of mRNA-Seq results by qRT-PCR.

Quantitative reverse transcription and polymerase chain reaction (qRT-PCR) was used to corroborate gene expression of selected genes with total RNA from three biological replicates and primers outlined in Table S2 and Table S3. The qRT-PCRs were carried out as described previously (3). Synthesis of cDNAs was carried out as described previously (4). Cycling parameters were 5 min 95 ºC followed by 40 cycles of 15 s at 95 ºC and 1 min at 60 ºC. Dissociation curves were drawn to ensure the absence of non-

specific amplification, and reaction efficiency and starting concentrations of template were calculated with LinRegPCR version 2016.0 as described (5).

AiiA purification, activity measurement, and QS inhibition. The *aiiA* gene was PCR-amplified, cloned into pET SUMO, replicated in *E. coli* One Shot® Mach1™-T1R, and expressed in *E. coli* BL21(DE3) One Shot® using the Champion™ pET SUMO Protein Expression System as recommended by the manufacturer (Invitrogen/Life Technologies, NY). Recombinant polyhistidine-containing AiiA protein was purified under native conditions using the ProBond™ Purification System (Novex/Life Technologies, NY) as recommended by the manufacturer with minor modifications. Briefly, the induced cells were harvested by centrifugation, suspended at 50-times the initial concentration in 0.01 mM KH_2PO_4 phosphate buffer pH 7.5, and lysed by passage through a French pressure cell press. The resulting cell-free extract was subjected to centrifugation to remove cell debris, filtered through 0.4-μm membrane, and dialyzed in 50 mM NaH_2PO_4-0.5 mM NaCl, pH 8, prior to loading onto the ProBond™ nickel-chelating resin column. Collected fractions with AiiA protein were dialyzed in 0.01 mM KH_2PO_4 pH 7.5, 0.2-μm sterile-filtered, and stored at -20 ºC until use.

AiiA specific activity units were determined by measuring reduction of AHL concentration after 4 h. Briefly, C_{10}-HSL was added to a concentration of 2 μM in 500 μL of TE buffer, pH 7.5, 10 μL of purified AiiA solution was added, and the solution was incubated for 4 h at 30 ºC. The specific activity of lactonase (pmol C_{10}-HSL consumed [μg protein]$^{-1}$ h^{-1}) was determined by measuring AHL remaining by bioassay (see materials and methods), and calculating the rate of reduction in C_{10}-HSL per μg AiiA

protein per hour. AiiA was heat-inactivated by incubating aliquots of purified AiiA in screw-cap tubes at 110 ºC for 30 min in a heating block.

For QS inhibition transcriptome and other experiments, a specific number of activity units of 0.2-µm-filtered AiiA lactonase was added in *N. winogradskyi* batch cultures every 24 h (QS-deficient). When culture cell density was less than an OD_{600} of 0.005 or NO_2^- concentration was greater than 57 mM, 48 activity units of AiiA lactonase per mL culture was added (approximately 0.28 µg protein mL^{-1}). When cell density was greater than OD_{600} of 0.005, and NO_2^- concentration less than 57 mM, 120 activity units of AiiA lactonase was added per mL culture (approximately 0.71 µg protein mL^{-1}). For transcriptome experiments, the same amount of heat-inactivated AiiA lactonase were added to *N. winogradskyi* control cultures (QS-proficient). Cells were harvested for RNA extraction and mRNA-Seq 4 h after AiiA lactonase treatment on day 3 of the experiment.

REFERENCES

1. **Sayavedra-Soto L, Ferrell R, Dobie M, Mellbye B, Chaplen F, Buchanan A, Chang J, Bottomley P, Arp D.** 2015. *Nitrobacter winogradskyi* transcriptomic response to low and high ammonium concentrations. FEMS Microbiol Lett **362:**1-7.
2. **Carlson JM, Chakravarty A, DeZiel CE, Gross RH.** 2007. SCOPE: a web server for practical *de novo* motif discovery. Nucleic Acids Res **35:**W259-264.
3. **Mellbye BL, Bottomley PJ, Sayavedra-Soto LA.** 2015. Nitrite-oxidizing bacterium *Nitrobacter winogradskyi* produces N-acyl-homoserine lactone autoinducers. Appl Environ Microbiol **81:**5917-5926.

4. **Schuster M, Lostroh CP, Ogi T, Greenberg EP.** 2003. Identification, timing, and signal specificity of *Pseudomonas aeruginosa* quorum-controlled genes: a transcriptome analysis. J Bacteriol **185:**2066-2079.

5. **Ruijter JM, Ramakers C, Hoogaars WM, Karlen Y, Bakker O, van den Hoff MJ, Moorman AF.** 2009. Amplification efficiency: linking baseline and bias in the analysis of quantitative PCR data. Nucleic Acids Res **37:**e45.
