# Supplementary figures and images for: Quorum Quenching of Nitrobacter winogradskyi Suggests that Quorum Sensing Regulates Fluxes of Nitrogen Oxide(s) during Nitrification
Source: mBio. 2016 Oct 25;7(5):e01753-16. doi: 10.1128/mBio.01753-16 (PMC5080386; doi:10.1128/mBio.01753-16)

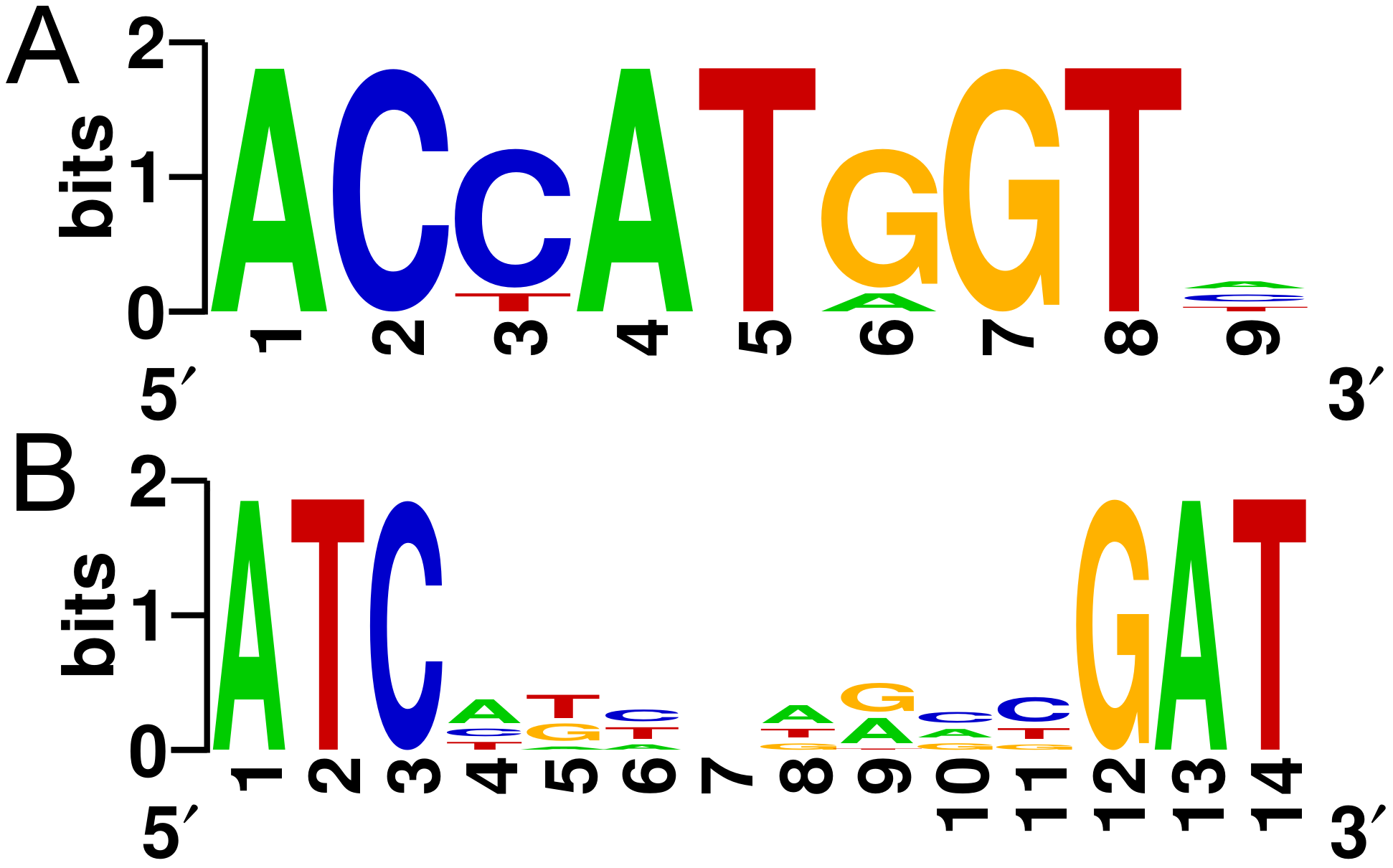

Supplement: Figure S1 — Putative lux-box-like promoter elements in N. winogradskyi. Sequence logos shown are graphic representations of aligned sets of putative promoter DNA sequences displaying frequencies of bases at each position as the relative heights of letters. The sequence logo also shows the degree of sequence conservation as the total height of a stack of letters, measured in bits. (a) Promoter motif A found upstream of nwiRI, the nirK cluster, and other genes. (b) Promoter motif B found upstream of nwiRI, the GppA phosphatase gene, and other genes. Table S2 shows all genes with these promoter motifs. Download [file mbo005163044sf1.tif]

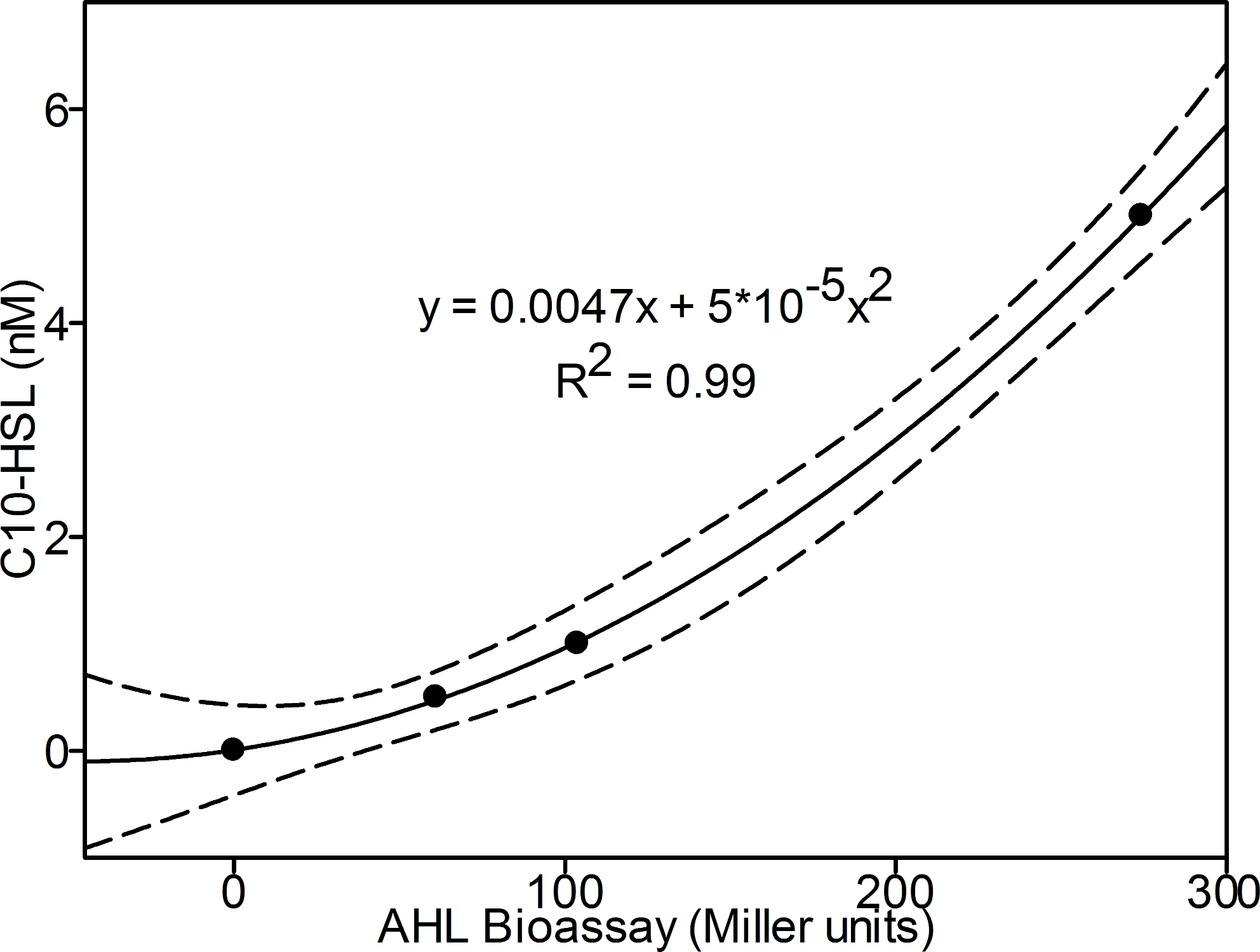

Supplement: Figure S2 — N-Decanoyl-l-homoserine lactone (C10-HSL) acyl-homoserine lactone (AHL) bioassay standard curve. Known concentrations of C10-HSL were added to bioassay cultures (see Materials and Methods in the main text) for estimation of the concentration of AHLs in N. winogradskyi cultures. The black line corresponds to the nonlinear regression of C10-HSL concentration (nanomolar, y axis) compared to AHL bioassay Miller units (x axis), and dotted lines indicate the 95% confidence band. The regression was calculated as follows: y = 0.0047x + 5 × 10− 5x2, R2 = 0.99. Download [file mbo005163044sf2.tif]
