# Supplementary material for: Quorum Quenching of Nitrobacter winogradskyi Suggests that Quorum Sensing Regulates Fluxes of Nitrogen Oxide(s) during Nitrification
Source: mBio. 2016 Oct 25;7(5):e01753-16. doi: 10.1128/mBio.01753-16 (PMC5080386; doi:10.1128/mBio.01753-16)
Supplement: Table S1 — Statistically significant changes in gene expression under QS-proficient conditions in N. winogradskyi [file mbo005163044st1.pdf]

Table S1. Statistically significant changes in gene expression during QS-proficient conditions in *N. winogradskyi*.

Gene number	Gene name	Role	Fold change[a]
Nwi0298, 0301, 0303		type II, III secretion/pilus assembly	-1.4 to -2.0
Nwi0317		intracellular septation protein A	-2.9
Nwi0529, 0597-0599, 1111, 1121-1124, 1130, 1132-1134	*flhA, fliH, fliG fliF*, *flgI*, *flgG*, *flgF*, *fliL*, *fliM*, *fliP*, *flgB*, *flgC*, *fliE*	flagella biosynthesis/assembly	-1.3 to -2.6
Nwi1119	*flgH*		1.8
Nwi0544-0547		secondary metabolite biosynthesis	1.5 to 2.3
Nwi0590, 0591		sulphite reductase subunits	-3.1, 1.3
Nwi0937		putative pigment protein	5.0
Nwi1031-1036	*fixK*	Crp/Fnr family, ABC transporter-associated loci	1.8 to 6.9
Nwi1153		Flp/Fap pilin component	1.8
Nwi1644, 1930, 2438		lytic murein transglycosylase	1.3 to 1.8
Nwi1667	*anmK*	anhydro-N-acetylmuramic	1.4

		acid kinase	
Nwi1913		arsenate reductase	-1.8
Nwi1939, 1940		CRISPR-Cas defense system	1.9, 1.6
Nwi2199		molybdenum cofactor cytidylyltransferase	-1.7
Nwi2348, 2349, 2710		protease FtsH	1.4, -1.3, 3.2
Nwi2403		teichoic acid and o-antigen export	-1.8
Nwi2410		O-antigen polymerase	-2.3
Nwi2761, 2763		sulfur metabolism	1.6, -1.7
Nwi2987, 2988		sulfur oxidation system SOX	2.4, 1.5
Nwi3022, 3023		sulfite dehydrogenase SorAB	4.3, 4.8
**Iron uptake & storage**			
Nwi0013	*fur*	ferric-uptake regulator	1.7
Nwi0035	*fur*	ferric uptake regulator	1.5
Nwi0700, 2049, 2318		TonB-dependent siderophore receptor	1.5, 2.0, -1.9
Nwi0895, 1339, 1969, 2297, 2882, 3080	*fecR*	iron dicitrate transport	1.6 to 2.4
Nwi1340, 1734,		σ^{70}, ECF subfamily, possible	2.1 to 14.5

1966, 2303, 2883, 3045, 3079		FecI	
Nwi2048-2052		FecI/FecR siderophore-associated loci	1.9 to 3.3
Nwi2075, 2322	*hmuV*	iron complex ABC transporter	-2.5, -3.3
Nwi2302		siderophore synthase component	2.4
Nwi2475, 2476		bacterioferritin	2.0, 4.8
Nwi2773		σ^{70}, ECF subfamily, possible FecI	-3.5
Nwi2975		ferrous iron transport	1.5
Nwi3050		iron complex ABC transporter-associated	-2.7
**Carbon metabolism**			
Nwi0033, 0034, 1243, 1244, 1407, 1686, 1690, 1753, 1849, 2993	*fabZ*	fatty acid metabolism	-1.3 to -2.9
Nwi0037		glyoxylate reductase	-1.6
Nwi0043, 0120, 1799	*coaD*	CoA metabolism	-1.5 to -2.3

Nwi0052, 0480, 1453, 1685, 2233		folate metabolism	-1.8 to -2.2
Nwi0054-0056	*trpA*	tryptophan metabolism	-1.5 to -1.9
Nwi0078, 0416		unsaturated lipid metabolism	1.7 to 2.1
Nwi0104, 0636, 0637, 2006, 2012, 2158, 2684, 3060	*coaE*	fatty acid metabolism	1.4 to 4.7
Nwi0122, 0124, 0126, 1330, 2390, 2565	*hisF*, *hisH*, *hisB, hisI, hisZ*	histidine biosynthesis	-2.1 to 1.37
Nwi0130, 1650, 2755		poly-β-hydroxybutyrate biosynthesis	1.3, 3.2, -1.9
Nwi0140, 0464 1862, 2351	*thyA*	pyrimidine metabolism	1.8 to 2.9
Nwi0153, 0208, 2007, 2008	*pyrEB, cmk*	pyrimidine metabolism	-1.9 to -4.9
Nwi0158, 0192, 1308, 1597, 2965	*purH*	purine metabolism	-1.5 to -2.5
Nwi0161, 0294, 2981		glutathione metabolism	1.4 to 2.9
Nwi0164, 0182, 0721, 1223, 1576		glutathione metabolism	-1.3 to -1.7
Nwi0201, 1410,	*dapF, dapD*	lysine metabolism	-1.4 to -2.2

2776, 3068, 3069			
Nwi0202, 0628, 0986, 1827, 1835, 2694, 2735, 2736	*pgm*, *pk*, *eno*, *pgk*	glycolysis/ gluconeogenesis	-1.3 to -2.2
Nwi0213, 2338		valine, leucine, isoleucine biosynthesis	1.6, 3.5
Nwi0229		threonine synthase	-1.4
Nwi0281		glycerol kinase	1.9
Nwi0283, 0284, 0403, 0586, 2890	*metH, metW*	methionine biosynthesis	1.5 to 7.7
Nwi0341	*mepA*	murein endopeptidase	1.5
Nwi0350		phosphoenolpyruvate carboxykinase	1.9
Nwi0379		aspartate kinase	-2.2
Nwi0395, 0396, 1224, 2977	*aroB*	phenylalanine, tyrosine, tryptophan biosynthesis	1.3 to 1.7
Nwi0423		2-oxoglutarate dehydrogenase E2	-1.4
Nwi0425, 1284-1286, 1813	*gcv*	Glycine cleavage system	-1.4 to -2.9
Nwi0467		acetyl-CoA synthetase	3.2
Nwi0495		farnesyl-diphosphate synthase	-1.5

Nwi0513, 0514, 2803, 3071	*argD*	arginine biosynthesis	-1.6 to -2.1
Nwi0566		hydroxypyruvate reductase	-1.9
Nwi0582, 1751		phenylalanine, tyrosine, tryptophan biosynthesis	-1.4, -2.0
Nwi0607, 1723	*ribH*	riboflavin metabolism	-1.4, -1.9
Nwi0629		chorismate synthase	-1.3
Nwi0633, 1855, 2592		terpenoid metabolism	1.4 to 1.7
Nwi0643		UDP-N-acetylglucosamine 4,6-dehydratase	-1.5
Nwi0696		phospholipid metabolism	-2.5
Nwi0702		4-hydroxymandelate oxidase	-1.3
Nwi0713		lysine metabolism	1.5
Nwi0752, 756, 2455		cobalamin adenosyltransferase	-1.4, -1.6, 2.6
Nwi0876		choloylglycine hydrolase	1.3
Nwi0924		ornithine decarboxylase	-2.2
Nwi0931, 1072, 1078, 1242, 1846, 1848, 1850, 2555, 2556	*lpxLBAD*	lipopolysaccharide biosynthesis	-1.3 to -2.6
Nwi0946, 1204-	*treXYZ*	starch/glycogen metabolism	-1.3 to -3.3

1210, 3024-3026			
Nwi1010, 1011		3-oxoacid CoA-transferase	2.1, 1
Nwi1014		phasin 2, PHA-binding	-2.0
Nwi1065		UDP-glucose 4-epimerase	1.5
Nwi1067, 1070		lipopolysaccharide biosynthesis	1.9
Nwi1079, 2204		putative CO dehydrogenase	4.1, 2.5
Nwi1085		aromatic metabolism	-1.5
Nwi1102		aromatic-L-amino-acid decarboxylase	-1.5
Nwi1213, 2159, 2160, 2236, 2237, 2537, 2601, 2791, 3018	*leuD*	valine, leucine, isoleucine degradation	1.3 to 3.9
Nwi1219, 1839, 1840, 2189, 2523	*moaAC*	molybdopterin biosynthesis	1.4, -1.4, -2.0, 1.9, 1.8, 3.5
Nwi1220		2-phosphoglycolate phosphatase	-1.5
Nwi1294	*idh*	isocitrate dehydrogenase	-1.5
Nwi1329, 1828	*folE*	folate metabolism	1.5, 1.6
Nwi1401, 1736		propionyl-CoA carboxylase beta chain	1.7, 1.4
Nwi1406		glycerol-3-phosphate	-1.8

		acyltransferase	
Nwi1420, 2688		homoserine metabolism	2.4, -1.8
Nwi1451		succinate semialdehyde dehydrogenase	1.5
Nwi1517, 2973		glucosamine metabolism	-2.8, -1.5
Nwi1525		cysteine synthase A	1.3
Nwi1530, 3067		cysteine metabolism	-1.9, -1.8
Nwi1571		guanine metabolism	1.3
Nwi1578		3-hydroxydecanoyl-[acyl-carrier-protein] dehydratase	1.5
Nwi1585		polysaccharide deacetylase	1.8
Nwi1604, 1605		carbamoyl-phosphate synthase	1.9, 2.2
Nwi1214, 1610, 1654		glyoxalase/ bleomycin resistance protein/dioxygenase	1.6 to 3.0
Nwi1646, 1745, 1817, 1818, 2641, 2737	*glpX*	glycolysis/ gluconeogenesis	1.3 to 2.7
Nwi1651		ornithine biosynthesis	-1.3
Nwi1721, 2465, 2466	*thiG*	thiamine biosynthesis	-1.6 to -4.3
Nwi1724		riboflavin metabolism	1.5

Nwi1739,		alcohol dehydrogenase	1.5
Nwi1766, 2272		phytoene biosynthesis	-2.5, -1.6
Nwi1787		putative β-glucans synthase	1.9
Nwi1805, 2309, 2646		glycoside hydrolase	-1.3 to -1.9
Nwi1837	*trpD*	tryptophan metabolism	1.6
Nwi1845		citrate synthase	1.6
Nwi1853, 2593		terpenoid biosynthesis	-1.4, -1.7
Nwi1873, 2420, 2421	*bioD*	biotin metabolism	-2.1 to -2.5
Nwi1904		glutamine synthetase	-2.8
Nwi1921		pyridoxl phosphate biosynthesis	-1.4
Nwi1947		D-xylulose 5P/D-fructose 5P utilization	1.9
Nwi1948		acetate kinase	2.7
Nwi1981-1985		carboxysome structural polypeptide	-1.7 to -2.6
Nwi1986		RuBisCo subunits	-2.2
Nwi1988		RuBisCO-associated LysR transcriptional regulator	-1.5
Nwi2014		alanine racemase	1.6
Nwi2024	*panB*	pantothenate metabolism	1.5

Nwi2067		putative L-sorbosone dehydrogenase	-1.8
Nwi2156, 2785		valine, leucine, and isoleucine metabolism	-1.6, -1.4
Nwi2218		ribulose-phosphate 3-epimerase	-1.9
Nwi2262		benzoate metabolism	-1.7
Nwi2263, 2516		Proline/Arginine metabolism	1.5, 5.9
Nwi2266, 2689	*ispH_1*, *ispH_2*	terpenoid metabolism	-1.5, 1.4
Nwi2321		glycine amidinotransferase	-1.8
Nwi2345, 2968, 2969		serine biosynthesis	-2.4, -1.9, -3.3
Nwi2353	*fumC*	fumarase	1.4
Nwi2378		SAM synthetase	2.5
Nwi2380		UDP-glucose dehydrogenase	1.4
Nwi2384, 2385		fructose metabolism	-1.4, -2.3
Nwi2396-2398		amino/nucleotide sugar metabolism	-1.6, 1.5, 1.9
Nwi2446, 2449	*carB*	carbamoyl-phosphate synthase	-3.0, -1.4
Nwi2468		thiamine biosynthesis	1.5
Nwi2514		proline metabolism	-2.7
Nwi2520		valine, leucine, and	-1.7

		isoleucine metabolism	
Nwi2552, 2888		purine metabolism	1.3, 1.3
Nwi2579		glycolate oxidase iron-sulfur subunit	1.9
Nwi2697		pyridoxal phosphate biosynthesis	-1.9
Nwi2734		transketolase	-1.3
Nwi2740		inositol monophosphatase	-1.7
Nwi2765		malate synthase	2.0
Nwi2792		carbonic anhydrase	1.4
Nwi2793		aspartate semialdehyde dehydrogenase	-2.7
Nwi2798-2800	*sdhA*	succinate dehydrogenase	1.3 to 1.9
Nwi2927, 2929	*rbcL_2*	CbbX and RuBisCo subunit	-2.6, 1.6
Nwi2947		N-formylglutamate amidohydrolase	1.8
Nwi2950		inositol monophosphatase	-2.0
Nwi2958		UDP-glucose pyrophosphorylase	-2.9
Nwi2980		aspartate aminotransferase	1.4
Nwi2985		isocitrate lyase	1.5
Nwi2990	*glpD*	glycerol-3-phosphate dehydrogenase	3.1

Nwi2992		acyl-CoA thioesterase	-1.5
**Nitrogen metabolism**			
Nwi0076, 0133, 1903		P-II, N regulation	2.2, -1.53, -3.8
Nwi0378		phosphoenolpyruvate phosphotransferase	-1.4
Nwi0384, 1302		nitrilase/cyanide hydratase, cyanase	2.0, -1.6
Nwi0557	*nnrS*	NO-related protein	8.1
Nwi0719, 0720	*nirBD*	assimilatory nitrite reductase	-2.5 to -9.3
Nwi0774, 0776-0778, 0965, 2068	*nxrAB*	nitrate reductase	3.8 to -2.2
Nwi0779	*nrt*	nitrite/nitrate transporter	-1.5
Nwi2061	*nnrR?*	Crp domain regulator	8.9
Nwi2653-2648	*nirK*	potential NO-producing/consuming gene cluster	2.2 to 19.9
Nwi1419	*nrt*	nitrite/nitrate transporter	2.4
Nwi1502		allophanate hydrolase subunit	2.2
Nwi1975		von Willebrand factor A	-2.1
Nwi2025		NnrU domain, NO	1.8

		reduction?	
Nwi2243		NosD accessory protein domain	1.4
Nwi3001		nitronate monooxygenase	3.1
**Energy transformation**			
Nwi0079, 0381, 0491, 1507	*ubiH*, *ubiG*	ubiquinone biosynthesis	1.4 to 3.0
Nwi0144		SCO1/SenC	-1.4
Nwi0166, 1878, 1879		NADH dehydrogenase	1.4 to 1.6
Nwi0191, 0977, 2388	*hemNEL*	heme metabolism	-1.7 to -2.6
Nwi0223, 0224, 0761, 0762	*coxB, coxA*	cytochrome c oxidase, subunit II, I	1.3 to 1.5
Nwi0225, 0763	*cyoE*	protoheme IX farnesyltransferase	-1.3 to -4.3
Nwi0228, 0766, 2313	*coxC, cocB*	cytochrome c oxidase, subunit III	-1.3, -2.4
Nwi0235-0239, 0428-431		ATP synthase	1 to -3.7
Nwi0242		heme metabolism	1.5
Nwi0323		aconitate hydratase	2.1

Nwi0328		amylo-alpha-1,6-glucosidase	1.9
Nwi0471, 1909	*hemCB*	heme metabolism	1.3. 1.8
Nwi0690, 0691		quinone biosynthesis	-1.8, -1.7
Nwi0753, 0754		electron transport flavoprotein	-1.6, -1.4
Nwi0764	*coxF*	cytochrome-associated	-1.5
Nwi0770	*cyoB*	cytochrome c oxidase subunit	2.2
Nwi0995-0997	*pnt*	alanine dehydrogenase/NAD(P) transhydrogenase	1 to -2.2
Nwi1094		phosphoglycerate/ bisphosphoglycerate mutase	2.0
Nwi1191, 1193, 2214	*cycH/ccmH*, *ccmF*	cytochrome biogenesis	-1.7, 1.2, -2.3
Nwi1335	*ppnK*	NAD^+ kinase	1.8
Nwi1504, 2750		NADPH:quinone reductase	3.3, 2.2
Nwi1594		polyphosphate kinase	1.4
Nwi1606		ETC complex 1 subunit	1.5
Nwi1659, 1663-1665, 1809		Fe-S cluster assembly	1.5 to 3.4
Nwi1661, 1662		Fe-S cluster assembly	-1.3, -1.6
Nwi1760		NADH-ubiquinone	2.1

		oxidoreductase	
Nwi1820		NADPH:FMN reductase	-1.4
Nwi1876, 1880, 1882, 1883, 1992		NADH dehydrogenase	-1.3 to -2.6
Nwi2279		cytochrome P450	2.4
Nwi2423-2427	*nadA*	NAD biosynthesis	1.4 to 5.4
Nwi2616		cytochrome bc1	1.4
Nwi2656		K^+/H^+ antiporter subunit	-1.4
Nwi2659		Na^+/H^+ antiporter subunit	1.6
**Translation**			
Nwi0012		acyltransferase, protein modification	1.8
Nwi0024-0027, 0066, 0067, 0068, 0091, 0156, 0206, 0216, 0312, 0376, 0406, 0439, 0497, 0631, 0632, 0983, 0999, 1287, 1316, 1346-1349, 1359-1361, 1386, 1387, 1389, 1422, 1433, 1588, 1673, 1691,	*infB*, *rbfA*, *truB*, *rpsO*, *pheS, rplT*, *rpmI*, *leuS, rpsA*, *truB*, *rpsO*, *infA,* *rpmB*, *rplU, alaS,* *rpmF*, *rpmJ*, *rpsU*, *rpsD, rplK,* *rplA, rplJ, rplL,* *rpsL, rpsM, rplQ,* *rpsI, rpmG, aspS,* *rpsF*, *rpsR, nusB,*	translation-associated factors and ribosomal proteins	-1.3 to -12.1

1692, 1715, 1722, 1776, 1844, 1858, 1859, 1865, 2020, 2027, 2144, 2277, 2344, 2367, 2512, 2533, 2550, 2551, 2586, 2662, 2711, 2747, 2781-2783, 3143	*gltX, tsf, rpsB*, *engA*, *hisS, glyS,* *rpmE*, *rimM*, *trmD*, *rplS, rpsT*		
Nwi0440, 0975, 1228, 1362, 1363, 1367-1373, 1377-1383, 1642, 1694, 1843, 1856, 1911, 2547, 2817	*rpmA, cysS, rpsJ, rplB, rpsS, rplV, rpsC, rplP, rpsQ, rpsN, rplF, rplR, rpsE, rpmD, rplO*, *rplI*	translation-associated factors and ribosomal proteins	1.3 to 2.9
Nwi0971		methionine sulfoxide reductase A	-1.6
Nwi1750		DsbA oxidoreductase	-1.6
Nwi1796	*queA*	queuosine biosynthesis	-3.5
Nwi1798, 1836, 2171		peptidyl-proline isomerase	1.3 to 1.6
Nwi1821		isoprenylcysteine carboxyl methyltransferase	1.9

Nwi1900	*tig*	trigger factor	-3.8
Nwi2511		peptidyl-tRNA hydrolase	-9.4
Nwi2704		tRNA-modifying YgfZ	1.7
Nwi2978		elongation factor 2/G	5.3
Nwi3065	*fmt*	methionyl-tRNA formyl transferase	-2.0
Nwi3066	*truA*	tRNA pseudouridine synthase	-2.4
**DNA replication & repair**			
Nwi0003, 0367, 1280	*recFRA*	homologous recombination & repair	-1.4 to -2.3
Nwi0004	*gyrA*	DNA gyrase	3.0
Nwi0028		exoribonuclease	-1.6
Nwi0050		DNA repair helicase	-1.3
Nwi0085, 0203, 0205		base excision repair-associated	-1.6 to -2.7
Nwi0093, 0369	*holA*	DNA pol. III subunits	-1.9, -1.3
Nwi0094		chromosome partitioning	-1.4
Nwi0193	*rph*	ribonuclease PH	-2.7
Nwi0335	*radC*	DNA repair protein	1.8
Nwi0353	*ligD*	DNA ligase	-1.8
Nwi0357		DNA pol. I	1.4

Nwi0462		DNA ligase I	1.6
Nwi0532		plasmid segregation ATPase	1.5
Nwi1255		type I restriction enzyme	1.5
Nwi1392		recombination MgsA	-1.8
Nwi1464		thymidylate kinase	-1.6
Nwi1513		nucleotide excision repair	-1.5
Nwi1566		single-stranded binding protein	1.7
Nwi1589		ribonuclease D	-2.1
Nwi1601	*ndk*	nucleotide diphosphate kinase	-2.2
Nwi1683	*gmk*	guanylate kinase	-1.8
Nwi1712		DNA topoisomerase I	-1.5
Nwi1713		RNase R	-1.3
Nwi1720		RNase E	-1.4
Nwi1808		dGTPase	2.5
Nwi1810		AP endonuclease	3.1
Nwi1833	*pyrG*	CTP synthase	-1.9
Nwi1857	*pyrH*	uridylate kinase	-2.0
Nwi1860	*dnaE*	DNA pol. III subunits	1.7
Nwi1872		RNase J	-2.2
Nwi1892		DNA-binding protein HU-beta	1.4

Nwi1914		DNA topoisomerase IV subunit A	-2.3
Nwi1915	*recO*	homologous recombination	1.5
Nwi1925		uracil-DNA glycosylase superfamily	-1.7
Nwi2004		Holliday junction resolvase	-1.5
Nwi2010		DNA processing, SMF	-1.5
Nwi2143	*guaA*	GMP synthase	-2.7
Nwi2146		IMP synthase	-2.3
Nwi2226		ATPase, DNA replication	-1.9
Nwi2336		resolvase	-3.7
Nwi2241	*dnaG*	DNA primase	1.3
Nwi2447		Dps, starvation-inducible DNA-binding	-1.7
Nwi2529		ribonuclease T2	2.5
Nwi2548, 2553	*mutL, xseA*	DNA mismatch repair	2.3, 1.8
Nwi2598	*rnhB*	RNase HII	1.4
Nwi2605		A/G-specific glycosylase	1.7
Nwi2724, 2725	*ruvBA*	recombination proteins	1.4
Nwi3070		pyrimidine 5-nucleotidase	-3.5
Nwi3075		ribonuclease P	-5.0
**Cell cycle**			

Nwi0525, 1200, 1431, 1432	*ctrA, pleC, divK, pleD*	cell cycle regulation	1.5 to 1.8
Nwi1042		peptidoglycan amidohydrolase	1.9
Nwi1045-1047, 1051-1055, 1716	*pbpB*, *murE*, *murG*, *murC*, *murB*	peptidoglycan biosynthesis	-1.3 to -2.4
Nwi1057, 1058	*ftsA*, *ftsZ*	cell division proteins	1.4
Nwi1138	*cckA*	PAS domain, Cell cycle	-1.3
Nwi1150, 1772		peptidoglycan biosynthesis	2.2, 1.5
Nwi1804		condensing subunit ScpA	-1.5
Nwi1978		chromosome partitioning	-2.3
Nwi2859, 2933		ParB-like nuclease	-2.1, 1.5
Nwi2946	*cpdR*	cell cycle regulation	-1.4
Nwi3073	*engB*	GTP-binding, chromosome partitioning	-1.4
**Stress**			
Nwi0008, 0952, 1024, 2683	*uspA*	universal stress protein	1.6 to 2.7
Nwi0030		heme catalase/peroxidase	-1.5
Nwi0129	*hslU*	heat shock protein HslU	-2.1
Nwi0189		heat shock metallopeptidase	2.6
Nwi0195	*grpE*	chaperone	-1.4

Nwi0197	*dnaK*	heat shock protein Hsp70	1.4
Nwi0211, 2952		heat shock protein Hsp20	2.6, 4.0
Nwi0442	*obgE*	GTP1/OBG domain GTPase	2.5
Nwi0589, 1695, 1696, 1898	*clpBA*, *clpS, clpX*	Clp protease	1.2 to 3.2
Nwi1113	*dnaK*	chaperone	-2.0
Nwi1195, 1897		serine protease	1.6, 1.8
Nwi1458		alkylhydroperoxidase	3.7
Nwi1508		DNA-binding, HspQ	-2.1
Nwi1593		stringent response	-1.6
Nwi1599, 1609, 2375, 3054		cold-shock protein CspA family	4.4, 1.6, -2.3, 2.1
Nwi1738		alkyl hydroperoxide reductase	1.6
Nwi1775	*surE*	phosphatase/nucleotidase	-1.6
Nwi1841		LexA SOS repressor	-1.5
Nwi1922		RelA/SpoT homolog	2.3
Nwi2151		Ppx/GppA phosphatase	3.2
Nwi2191, 2192, 2574	*groES_2, groEL_2, groEL_3*	chaperonins	-1.5 to -1.9
Nwi2280		OsmC domain	1.6
Nwi2320		multidrug resistance	-2.5

Nwi2796		Cu-Zn superoxide dismutase	-1.5
Nwi2812		Hsp90	1.4
Nwi2940		Hsp20	-3.6
**Transcription**			
Nwi0100	*rho*	transcription term.	-3.7
Nwi0111	*regA*	redox response	2.7
Nwi0177, 0178		σ^{54} & activation protein	1.6, 4.2
Nwi0194	*hrcA*	negative regulator of heat shock protein	2.4
Nwi0348		acidity-sensing regulator	-1.4
Nwi0500, 0982, 2647		diguanylate cyclase/phosphodiesterase	3.7, 1.7, -2.1
Nwi0565		MarR-like regulator	-2.0
Nwi0626	*nwiI*	autoinducer synthesis	2.5
Nwi0627	*nwiR*	AHL-binding LuxR	1.3
Nwi0750		ribonucease BN	1.9
Nwi0893		σ^{24}, ECF subfamily	2.7
Nwi0922	*greAB*	transcription elongation factor	2.0
Nwi0957		LuxR family regulator	2.3
Nwi1309		BolA family regulator	1.7
Nwi1345	*nusG*	transcription antiterminator	-4.7
Nwi1351		RNA polymerase $\beta^{'}$ subunit	1.4

Nwi1388		RNA polymerase α subunit	-2.3
Nwi1409		MerR domain regulator	2.4
Nwi1444, 1446	*glnL, ntrY*	N availability, two-component system	1.9, -2.1
Nwi1449	*hfq*	regulatory RNA factor	2.6
Nwi1740		adenylate/guanylate cyclase	-1.9
Nwi2430	*rpoH*	σ^{32}, heat shock sigma factor	1.3
Nwi2440	*rpoD*	σ^{70}, primary sigma factor	1.9
Nwi2907-2909		ArsR loci	2.8 to 6.6
Nwi3032		Crp/Fnr transcriptional regulator	2.3
Nwi3059		polyhydroxyalkanoate synthesis repressor PhaR	1.4
**Transport**			
Nwi0106, 0390 1344, 1769, 1770, 1834, 2779, 3074	*secBAEDG*, *yajC*, *yidC*	Sec Type II secretion	-1.4 to -4.5
Nwi0173	*lptA*	lipopolysaccharide export	1.7
Nwi0150		polar amino acid ABC transporter	-1.5
Nwi0288-0292		small peptide ABC transporter	-1.6 to 1.4
Nwi0329	*oprB*	carbohydrate-selective porin	-1.4

Nwi0331-0333		NitT/TauT ABC transporter	1 to -1.9
Nwi0337		K^+/H^+ antiporter	2.1
Nwi0338-0340	*modABC*	molybdate ABC transporter	1 to -3.7
Nwi0356		lysine exporter	-2.4
Nwi0458		NitT/TauT ABC transporter related	2.3
Nwi506-509	*pst*	phosphate transport	1 to 2
Nwi0606, 2853		Na^+/H^+ transporter	-2.1, 1.6
Nwi0611		phosphate ABC transporter	-2.0
Nwi0680-0683		ABC-type nitrate/sulfonate/ bicarbonate transport system	1.8 to 3.2
Nwi703, 0704	*exbBD*	biopolymer transport	1.5
Nwi0705	*tonB*	periplasmic TonB	-1.6
Nwi0780		C4-dicarboxylate transporter/malic acid transport	-2.8
Nwi0875	*tamA*	translocation and assembly, surface antigen	-2.9
Nwi0880		molybdenum ABC transporter	-2.2
Nwi0914-0916		zinc/manganese ABC transporter	-1.6 to -3.0
Nwi0935		MscS ion channel	-1.5

Nwi1000		aquaporin	4.8
Nwi1006, 1007, 1008, 1241		spermidine/putrescine ABC transporter	2.0, -1.3, -1.4, -2.3
Nwi1152, 1437, 3027		MFS_1	-1.4 to -2.6
Nwi1031, 1032		ABC transporter & hypothetical	6.9
Nwi1201		cation efflux protein	-1.9
Nwi1233, 2153		uncharacterized ABC transporter & related	-3.9
Nwi1236, 2305, 2720		MotA/TolQ/ExbB proton channel	1.6, -2.1, -1.4
Nwi1238		K^+ transporter	-1.5
Nwi1268, 1269, 2053, 2306		biopolymer transport ExbD/TolR, TonB	-1.5 to -2.3
Nwi1384	*secY*	Sec Type II secretion	1.3
Nwi1434		TolC-type OMP	1.3
Nwi1455-1457		heavy metal efflux	-1.9, -1.8, 1.4
Nwi1503		LamB/YscF porin-type	1.8
Nwi1510		peptide/nickel ABC transport system	2.0
Nwi1527-1528, 2892		amino acid ABC transporter	-1.5, -1.7, 1.4

Nwi1777-1779	*tatCBA*	twin-arginine translocation protein	1.6 to 2.5
Nwi1863, 1864	*lolDCE*	lipoprotein ABC transporter	1.5, 1.3
Nwi2031		phosphate transporter	-1.4
Nwi2065		sulfate permease, SulP	1.6
Nwi2169		phosphate-selective porin	-1.8
Nwi2209, 2211-2213		branched-chain amino acid ABC transporter	1.8, -2.6, -1.6, 2.0
Nwi2219		vitamin B12 TonB-dependent receptor	-1.4
Nwi2240		ArgK domain	1.9
Nwi2284		secretion protein HlyD	1.7
Nwi2340		glycine betaine ABC transport system	-5.1
Nwi2361, 2362		ABC-2 type transporter	-1.6, -1.5
Nwi2631		TonB-dependent Sugar transporter family	-2.5
Nwi2681		glucan exporter	-1.8
Nwi2717	*tolB*	Tol-dependent translocation	-1.3
Nwi2728		divalent cation transporter	-1.5
Nwi2741		multidrug efflux pump	-1.3
Nwi2757-2759		sulphate transport system permease	-1.4 to -1.6

Nwi2911-2912	heavy metal efflux pump	-2.2
Nwi3006	formate/nitrite transporter	1.4
**Hypothetical, general function only, & mobile elements**		
Nwi0006, 0272, 0275, 0308, 0310, 0365, 0794, 0834, 0954, 0976, 1016, 1140-1142, 1172, 1278, 1291-1293, 1297, 1321, 1322, 1358, 1397, 1490, 1565, 1633, 1718, 1999, 2260, 2411, 2437, 2821, 2838, 2918-2922, 2949, 3008, 3058, 3138	transposon or phage-related	1.4 to 4.2
Nwi0007, 0147, 0214, 0435, 0730-0737, 0781, 0792, 0838, 0972, 1173,	transposon or phage-related	1 to -12.0

1282, 1472, 1482,		
1488, 1572, 1591,		
1622, 1626, 1629,		
1934, 1950, 2108,		
2119, 2120, 2142,		
2370, 2474, 2498,		
2499, 2818, 2923,		
2925, 2944, 2945,		
3013, 3037, 3038,		
3115		
Nwi0009, 1328	NifU-like	4.4
Nwi0014, 2258	MiaB-like	1.6, -1.6
Nwi0015	PhoH-like	2.2
Nwi0016-0017,	transporter-associated	-1.4 to -2.3
0160, 0397		
Nwi0018	acyltransferase	-1.8
Nwi0019, 0087,	transcriptional regulator	2.3 to 2.5
Nwi0020, 1894,	methyltransferase	1.4 to 2.1
1896		
Nwi0029	transcriptional regulator	-2.1
Nwi0032	dehydrogenase/ reductase	-1.7
Nwi0036, 0039,	hypothetical	1.2 to 18.6
0047, 0061, 0062,		

0075, 0107, 0112,

0131, 0143, 0155,

0168, 0190, 0196,

0212, 0220, 0256,

0257, 0263, 0264,

0325, 0354, 0372,

0374, 0382, 0383,

0391, 0413, 0437,

0450, 0454, 0455,

0459, 0461, 0463,

0465, 0468, 0473,

0474, 0476, 0486,

0515, 0516, 0518,

0527, 0528, 0531,

0561, 0573, 0578,

0581, 0600, 0609,

0625, 0650-0652,

0663, 0687, 0710,

0740-0745, 0760,

0767, 0768, 0772,

0785, 0786, 0814,

0837, 0839, 0841,

0863, 0878, 0889,

0893, 902, 0906,

0909, 0910, 0927,

0936, 0944, 0945,

0955, 0959, 0966,

0989, 0994, 0998,

1003, 1004, 1084,

1086, 1092, 1108,

1145-1148, 1165,

1166, 1169, 1170,

1197, 1222, 1232,

1247, 1248, 1256,

1257, 1260, 1264,

1270, 1273, 1275,

1276, 1303, 1327,

1336, 1356, 1417,

1428, 1467, 1499,

1501, 1512, 1548,

1569, 1570, 1580,

1581, 1586, 1602,

1603, 1610, 1613,

1634-1637, 1639,

1640, 1660, 1701,

1703, 1704, 1728,

1742, 1759, 1762,

1763, 1783, 1785,

1791, 1822, 1823,

1867, 1895, 1902,

1972, 1993, 2033,

2058, 2063, 2064,

2147, 2149, 2161,

2163, 2166, 2190,

2194, 2196, 2200,

2201, 2231, 2232,

2239, 2244, 2256,

2257, 2264, 2268,

2281, 2304, 2337,

2352, 2355, 2356,

2369, 2379, 2399,

2414, 2415, 2418,

2422, 2436, 2439,

2456, 2459, 2469,

2480, 2489, 2493,

2494, 2497, 2500,

2502, 2503, 2505,

2530-2532, 2575,

2576, 2578, 2588,

2591, 2602, 2625, 2660, 2680, 2685, 2707, 2715, 2721, 2732, 2733, 2743, 2748, 2752, 2788, 2789, 2794, 2807, 2808, 2813-2815, 2835, 2837, 2849, 2870, 2871, 2884, 2889, 2893, 2894, 2900, 2955, 2956, 2983, 2984, 2989, 2991, 3002, 3041, 3047, 3082, 3108, 3128, 3137, 3140-3142		
Nwi0045, 0559, 1012, 1087, 1198, 1744, 3077	histidine kinase	1.3 to 3.2
Nwi0046, 0064, 0077, 0080, 0092, 0142, 0146, 0167, 0183, 0210, 0234,	hypothetical	-1.3 to -15.0

0250, 0260, 0262,

0267, 0295, 0313,

0346, 0368, 0375,

0398, 0402, 0405,

0424, 0438, 0447,

0498, 0499, 0534,

0562, 0577, 0587,

0610, 0612, 0646,

0718, 0757, 0873,

0884, 0886, 0887,

0901, 0904, 0905,

0907, 0918, 0920,

0930, 0932, 0939,

0940, 0967, 0970,

0985, 0987, 0990,

1021, 1022, 1039,

1041, 1090, 1105,

1107, 1114, 1125,

1126, 1128, 1139,

1154, 1155, 1185,

1202, 1288, 1307,

1310, 1313, 1331,

1332, 1352, 1355,

1390, 1405, 1454,

1468, 1470, 1494,

1519, 1520, 1547,

1550, 1559, 1600,

1631, 1666, 1672,

1684, 1689, 1693,

1698, 1705, 1767,

1792, 1812, 1815,

1869, 1891, 1908,

1916, 1924, 1936,

1937, 1946, 1973,

1974, 1979, 1990,

1991, 2011, 2021-

2023, 2032, 2054,

2070, 2087, 2145,

2152, 2173, 2177,

2197, 2208, 2220,

2230, 2265, 2275,

2291, 2316, 2334,

2347, 2373, 2389,

2402, 2404, 2409,

2432, 2458, 2472,

2482-2484, 2510,

2513, 2528, 2557, 2558, 2585, 2597, 2635-2637, 2667, 2669, 2690, 2691, 2702, 2730, 2749, 2756, 2767-2770, 2772, 2775, 2784, 2786, 2806, 2826, 2868, 2869, 2872, 2873, 2876, 2878, 2901, 2915, 2935, 2943, 2959, 2966, 2970, 2979, 3021, 3035, 3042, 3072, 3078, 3101, 3107, 3118-3121		
Nwi0059, 0074, 0221, 0448, 0452, 0466, 0926, 0993, 1271, 1274, 2170, 2184, 2346, 2368, 2545, 2846, 3029	peptidase	1.3 to 3.5
Nwi0063, 2270	amine oxidase	1.5, -1.7

Nwi0073, 0716	thioredoxin	1.5 to 1.7
Nwi0103	Maf-like	2.3
Nwi0108	transglycosylase	1.7
Nwi0109	Smr/MutS2	-2.0
Nwi0113, 0653, 0864, 0890, 2288, 2895	beta-lactamase-like	1.3 to 3.8
Nwi0114, 0119, 1754, 2774	histidine kinase	-1.5 to -2.0
Nwi0118	Mg chelatase-related	1.4
Nwi0138	phospholipase	1.5
Nwi0141	camphor resistance	-2.8
Nwi0145, 0552, 1413, 2112, 2195	glycosyl transferase	-1.6 to -2.0
Nwi0149, 0541	glycosyl transferase	1.4
Nwi0157	Heparinase-like	1.6
Nwi0186	metallophosphoesterase	1.7
Nwi0187	Lhr family helicase	1.4
Nwi0188, 0244, 1337, 1567, 1832, 2527, 2845	putative outer membrane protein (OMP)	1.4 to 2.3
Nwi0207, 0386	methyltransferase	-1.5 to -1.8
Nwi0218	PfkB kinase	-2.0

Nwi0219, 0616	pirin domain	2.3 to 3.6
Nwi0567, 1391, 1671, 1749, 1919, 2471, 2934	peptidase	-1.4 to -1.9
Nwi0226	CoxF domain	-1.5
Nwi0241	LuxR domain, no AHL-binding domain	-1.6
Nwi0249	tyrosine phosphatase	-1.6
Nwi0253	siderophore receptor	1.7
Nwi0270	type I restriction enzyme, R subunit	2.6
Nwi0293, 2708	cell wall-associated hydrolase	1.4, -1.7
Nwi0314, 0359, 1657	DEAD/DEAH box helicase	-1.7 to -7.0
Nwi0336	methionine aminopeptidase	3.3
Nwi0343	GTP-binding, LepA homolog	-1.7
Nwi0349	pyridoxamine 5'-phosphate oxidase-related	3.8
Nwi0358, 0441, 0485	acyltransferase	1.9 to 2.9
Nwi0362	AsnC/Lrp transcriptional	-3.8

	regulator	
Nwi0385	phosphoribosyltransferase	2.9
Nwi0409	SUPV3L1/SUV3 helicase	-1.4
Nwi0411	ferredoxin	-2.7
Nwi0412	CarD family transcriptional regulator	1.6
Nwi0451, 2624, 2633	NUDIX hydrolase	1.6 to 2.0
Nwi0453	nuclease	-3.1
Nwi0553	HAD hydrolase	1.5
Nwi0554	GTP-binding protein	-1.9
Nwi0555	transcriptional regulator	-1.7
Nwi0615, 0934, 2454	LysR regulator	1.3 to 1.6
Nwi0619	L,D-transpeptidase domain	1.8
Nwi0621	SDR	1.3
Nwi0635	histone deacetylase	1.9
Nwi0642	aminotransferase	-1.7
Nwi0645	acylneuraminate cytidylyltransferase	-1.6
Nwi0648	NUDIX hydrolase	-1.6
Nwi0662	XRE family regulator	-1.7
Nwi0725, 0879,	TonB-dependent receptor	-1.5 to -2.9

0941, 1868		
Nwi0836, 1230	acetyltransferase	1.5, 1.3
Nwi0881	GntR family regulator	1.8
Nwi0882	class II aldolase/adducin	1.8
Nwi0891	peroxiredoxin	1.9
Nwi0892	ArsR family regulator	2.6
Nwi0898, 2401	NAD-dependent epimerase/dehydratase	1.4, -1.5
Nwi0918	WD-40 repeat protein	1.5
Nwi0949, 0950	kinase/Mo blast hit	2.4, 3.6
Nwi0980, 2140	LysR regulator	-2.3, -4.8
Nwi0984, 2713	TPR repeat	2.4, 1.4
Nwi1005, 1098, 1655, 2282, 2365, 2595	FAD-dependent oxidoreductase	1.4 to 3.2
Nwi1009	AsmA homolog, protein assembly	-2.9
Nwi1026	phosphoketolase	-2.2
Nwi1027	CheY-like	-2.1
Nwi1127	tetratricopeptide TPR_4	-1.6
Nwi1187, 1188	OmpR family regulator and histidine kinase	3.0, 2.3
Nwi1324	patatin-like, storage protein	-1.3

Nwi1343, 2461	PRC-barrel domain	-3.6, 1.7
Nwi1412	sugar transferase	-1.3
Nwi1441	putative CinA	-1.6
Nwi1500	cupin region	6.0
Nwi1509	IalB domain	1.5
Nwi1541	Fis-type domain	1.4
Nwi1583	CreA family	3.2
Nwi1590	hemolysin-like	1.5
Nwi1755, 2771	response regulator receiver	-2.6, -1.5
Nwi1761, 2018, 2632	acetyltransferase	-1.4 to -2.0
Nwi1765	serine/threonine kinase	-1.4
Nwi1774	response regulator receiver	1.6
Nwi1814, 2744, 2802, 3028	putative OmpA/MotB	-1.7, 1.3, -1.9, 6.2
Nwi1851	putative OMP	-1.3
Nwi1852	metallo peptidase	-1.3
Nwi1866	GLE1 domain	1.8
Nwi1901, 2660	multicopper oxidase	1.9, 1.9
Nwi1906, 2325	antibiotic biosynthesis monooxygenase domain	-1.4, -1.6
Nwi1933	putative porin	2.1
Nwi2016	putative colicin V production	-1.4

Nwi2019, 2310, 2393	SDR	-1.5 to -5.7
Nwi2071	luciferase-like	-3.5
Nwi2084, 2089, 2203, 2485, 2626	AAA domain, putative ATPase	-2.1, 2.3, 4.2, -2.2, 1.4
Nwi2178, 2207	alpha/beta hydrolase	-2.0, 1.9
Nwi2227	ROSMUCR transcriptional regulator	2.2
Nwi2276, 2451	putative oxidoreductase	-2.1, -1.5
Nwi2289	HpcH/HpaI aldolase domain	2.2
Nwi2290, 2507, 2508	MaoC-like dehydratase	1.9 to 2.4
Nwi2394	nucleotidyl transferase	-2.1
Nwi2395	hexapeptide transferase	-2.0
Nwi2417	cyclic nucleotide-binding	1.5
Nwi2418	Cu-binding hypothetical	15.9
Nwi2463	lytic transglycosylase	-1.9
Nwi2488, 2570, 2891, 3017	ErfK/YbiS/YcfS/YnhG	-2.0, 2.4, 1.7, 1.6
Nwi2517, 2518	OmpR family regulator & histidine kinase	-3.2, -1.6
Nwi2590	methyltransferase	2.0
Nwi2607	protein-disulfide isomerase	1.7

Nwi2609-2612	LemA loci	1.9 to 7.8
Nwi2679	DedA family	-2.7
Nwi2804	phosphoesterase	-1.9
Nwi2810	ferredoxin	1.4
Nwi2997	CsbD-like protein	-1.8
Nwi2998	RHodanese-like	-1.4
Nwi3009	PadR-like	1.7
Nwi3030	HlyD secretion protein domain	-1.3
Nwi3031	acriflavin resistance	-2.0
Nwi3033	AsmA, OM assembly	1.5
Nwi3083	AbrB family transcriptional regulator	2.0
Nwi3111	XRA family transcriptional regulator	1.7

[a]Fold change is the difference in mRNA transcript levels between AiiA-treated QS-deficient cells and QS-proficient cells ($P \leq 0.05$).
