# Supplementary material for: Quorum Quenching of Nitrobacter winogradskyi Suggests that Quorum Sensing Regulates Fluxes of Nitrogen Oxide(s) during Nitrification
Source: mBio. 2016 Oct 25;7(5):e01753-16. doi: 10.1128/mBio.01753-16 (PMC5080386; doi:10.1128/mBio.01753-16)
Supplement: Table S2 — Putative QS-controlled genes with upstream lux-box-like promoter elements. [file mbo005163044st2.pdf]

Table S2. Putative QS-controlled genes with upstream *lux*-box-like promoter elements.[a]

Motif[b]	Location, strand[b]	Gene number	Gene name	Role	Fold change[c]
<u>**A**</u>					
**acyatrgth**					
accatagta, actatggtc	-171 to -163, + -212 to -220, -	Nwi0627	*nwiR*	putative AHL-binding LuxR homolog	1.3
accatggtt, accatggtc	-266 to -274, - -273 to -265, +	Nwi0637	*coaE*	acyl-CoA dehydrogenase	1.8
accatggta, accatggtc	-92 to -100, - -99 to -91, +	Nwi0914		ABC transporter-related	-2.4
accatggtc, accatggtt	-218 to -226, - -225 to -217, +	Nwi1316	*rpsD*	ribosomal protein	-3.1
accatggtt, accatggta	-212 to -220, - -219 to -211, +	Nwi2653-2648[c]	*nirK*, *ncgABC*	potential NO-producing/ consuming gene cluster	2.2 to 19.9
<u>**B**</u>					
**atchdhnddvbgat**					

atccgattaatgat, atcatcggagtgat, atcttccaaacgat, atcctcatggtgat, atcattcatccgat	-104 to -91, + -15 to -2, + -407 to -420, - -316 to -329, - -56 to -69, - -233 to -220, +	Nwi0080, 0402, 0403, 1972, 2908, 3041		hypothetical	-2.1 to 6.6
atcagcttggcgat	-67 to -80, -	Nwi0627	*nwiR*	putative AHL-binding LuxR homolog	1.3
atccgtcaagcgat	-83 to -70, +	Nwi0781		transposase	-4.6
atcaatcggccgat	-52 to -39, +	Nwi1035		UspA, universal stress protein loci	3.0, 3.4
atctttcaacggat	-299 to -312, -	Nwi1286	*gcvT*	glycine cleavage system	-2.9
atcatccagcggat	-140 to -127, +	Nwi1386	*rpsM*	ribosomal protein	-3.7
atccgaaagacgat	-127 to -114, +	Nwi2151		Ppx/GppA phosphatase, stringent response	3.2
atcagtatgctgat	-51 to -38, +	Nwi2455		folic acid metabolism	2.6

atctttcagacgat, atccgataaatgat, atcatttatcggat, atctttcagacgat	-547 to -534, + -312 to -299, + -325 to -312, + -77 to -90, -	2512, 2513	hypothetical ribosome-associated	-5.7, -3.8, -3.0
atcaaaggaccgat	-443 to -430, + -246 to -259, -	Nwi2845, 2846	conserved peptidase C14	3.5
atctgcctgaggat	-307 to -294, +	Nwi3028	OmpA/MotB	6.2

[a]All motifs are also found in the intergenic region between Nwi0627 (*nwiR*) and Nwi0628 (*pgm*). No motifs were detected in the 81 bp intergenic space between Nwi0626 (*nwiI*) and *nwiR.*

[b]Sequences, upstream location, and strand are listed in the order of gene numbers in the gene number column. Some sequence motifs appear on both strands for a single gene or in front of multiple genes.

[c]Fold change is the difference in mRNA transcript levels between AiiA-treated QS-deficient cells and QS-proficient cells ($P \leq 0.05$).

[d]The motif was only found within intergenic region upstream of Nwi2653, the putative start site of the operon.
