# Supplementary material for: Quorum Quenching of Nitrobacter winogradskyi Suggests that Quorum Sensing Regulates Fluxes of Nitrogen Oxide(s) during Nitrification
Source: mBio. 2016 Oct 25;7(5):e01753-16. doi: 10.1128/mBio.01753-16 (PMC5080386; doi:10.1128/mBio.01753-16)
Supplement: Table S3 — Bacterial strains and plasmids. [file mbo005163044st3.pdf]

Table S3. Bacterial strains and plasmids.

Strain or plasmid	Relevant genotype or phenotype	Reference or source
Strains		
N. winogradskyi Nb-255	ATCC 25391	(1)
E. coli		
TOP10	F^- *mcrA* Δ (*mrr-hsdRMS-mcrBC*) φ80d*lacZ*ΔM15 *ΔlacX74 recA1 araD139* Δ(*ara-leu*) *7697 galU galk rpsL* (Str^R) *endA1 nupG λ-*	Life Technologies
BL21(DE3)	F^- *ompT* $hsdS_B$ ($r_B^- m_B^-$) *gal dcm* (DE3)	Life Technologies
Plasmids		
pDSK519	Empty vector, Kan^R	(2)
pDSK-*aiiA*	Vector carrying *aiiA* from *Bacillus* 240B1, Kan^R	(2)
pET SUMO	TA-cloning, T7-regulated expression vector,	Life

	Kan^R	Technologies
pET SUMO-*aiiA*	Expression vector carrying *aiiA*, Kan^R	This study

REFERENCES

1. **Starkenburg SR, Chain PS, Sayavedra-Soto LA, Hauser L, Land ML, Larimer FW, Malfatti SA, Klotz MG, Bottomley PJ, Arp DJ, Hickey WJ.** 2006. Genome sequence of the chemolithoautotrophic nitrite-oxidizing bacterium *Nitrobacter winogradskyi* Nb-255. Appl Environ Microbiol **72:**2050-2063.
2. **Gao M, Chen H, Eberhard A, Gronquist MR, Robinson JB, Connolly M, Teplitski M, Rolfe BG, Bauer WD.** 2007. Effects of AiiA-mediated quorum quenching in *Sinorhizobium meliloti* on quorum-sensing signals, proteome patterns, and symbiotic interactions. Mol Plant Microbe Interact **20:**843-856.
