# Supplementary material for: Quorum Quenching of Nitrobacter winogradskyi Suggests that Quorum Sensing Regulates Fluxes of Nitrogen Oxide(s) during Nitrification
Source: mBio. 2016 Oct 25;7(5):e01753-16. doi: 10.1128/mBio.01753-16 (PMC5080386; doi:10.1128/mBio.01753-16)
Supplement: Table S4 — Comparison of fold changes in expression between QS-deficient and proficient treatments analyzed by quantitative PCR (qPCR) and mRNA-Seq. [file mbo005163044st4.pdf]

Table S4. Comparison of fold change expression between QS-deficient and proficient treatments analyzed by qPCR and mRNA-Seq.

Gene; name	mRNA-Seq	qPCR
Nwi0626; *nwiI*	2.5	2.2
Nwi0627; *nwiR*	1.3	1.2
Nwi2648; *nirK*	2.2	2.0
Nwi2650; *ncgB*	19.9	18.4
Nwi0557; *nnrS*	8.1	12.0
Nwi2061; Crp domain	8.9	6.4
