# Supplementary material for: Quorum Quenching of Nitrobacter winogradskyi Suggests that Quorum Sensing Regulates Fluxes of Nitrogen Oxide(s) during Nitrification
Source: mBio. 2016 Oct 25;7(5):e01753-16. doi: 10.1128/mBio.01753-16 (PMC5080386; doi:10.1128/mBio.01753-16)
Supplement: Table S5 — Genes and primers used to corroborate gene expression. [file mbo005163044st5.pdf]

Table S5. Genes and primers used to corroborate gene expression.

Gene name	Forward primer	Reverse primer
Nwi0626;	Nwi0626F:	Nwi0626R:
nwiI	ACGAGATGGAGCAGGCTTAT	AGAACGCGATCTTCTTCGAT
Nwi0627;	Nwi0627F:	Nwi0627R:
nwiR	TTCCTATTCCAGGGATGACC	GGTAGGGTCATCGAACAGGT
Nwi2648;	Nwi2648F:	Nwi2648R:
nirK	AATCCGAAGAACGTGATGGT	GGTGAGATGAGGTCGAAGGT
Nwi2650;	Nwi2650F:	Nwi2650R:
ncgB	AACTTGATCCGAGCTGTTCC	CCAGCCATTCCTTCTTGAAC
Nwi0557;	Nwi0557F:	Nwi0557R:
nnrS	CGATCATCGTTCTGATCTCG	AAGCATTCTGCCTGATGAGC
Nwi2061;	Nwi2061F:	Nwi2061R:
Crp domain	GATCGTCGGACTTCAATTCG	TTATGCTGCAGACCTCAACG
